# Niche-derived exosomes control *Drosophila* immune stress hematopoiesis

**DOI:** 10.3389/fimmu.2026.1824544

**Published:** 2026-06-10

**Authors:** Nathalie Vanzo, Marianne Montemurro, Christian Rouvière, Léonie Gargouil, Vanessa Soldan, Michèle Crozatier

**Affiliations:** 1 University of Toulouse, CNRS, CBI, Toulouse, France; 2 Agronutrition, Labège, France

**Keywords:** *Drosophila* innate immunity, extracellular vesicles, hematopoietic niche, lymph gland, parasitism

## Abstract

Hematopoietic stem and progenitor cells in the bone marrow ensure continuous blood cell production. Homeostasis is controlled by a specialized microenvironment termed the niche. Extracellular vesicles released by the hematopoietic niche, both under normal and stress conditions, regulate stem cell maintenance, proliferation and differentiation. Here, we show that the *Drosophila* hematopoietic niche in the larval immune organ, the lymph gland, releases extracellular vesicles that mediate cell communication necessary to adapt blood cell progenitor homeostasis to immune stress. In response to wasp parasitism, ROS elevation in the niche causes the release of a heterogeneous population of EVs, including exosomes, which propagate and activate non-cell autonomously the EGFR pathway in lymph gland progenitors. Niche-derived exosomes promote progenitor differentiation into lamellocytes, a blood cell type dedicated to the neutralization of the pathogen. We further show that up-regulation of the metalloproteinase Mmp1 that occurs in niche cells in response to wasp infestation is required for exosome spreading through the niche’s extracellular matrix. Our work establishes the *Drosophila* lymph gland as a novel *in vivo* model to study exosome-mediated cell communication and provides new insights into how niche-derived exosomes control immune stress hematopoiesis.

## Introduction

In the mammalian bone marrow, hematopoietic Stem and Progenitor Cells (HSPCs) ensure continuous blood cell production throughout adulthood. The decisions of HSPCs to self-renew, be quiescent or differentiate are tightly controlled by their micro-environment, called the niche (1–3). While our knowledge of HSPC/niche communication under homeostatic conditions progresses, processes involved during stress hematopoiesis remain more elusive. Recent data indicate that niche cells can sense infection or chronic inflammatory signals produced during normal aging, neoplastic tumors or bone marrow failure syndromes (2, 4, 5). Understanding how the niche controls HSPCs during stress physiology is a major challenge, as imbalanced niche signal production can alter quiescence and retention of HSPCs within the niche, as well as blood cell lineage outputs.

Extracellular vesicles (EVs) have emerged as prominent actors in niche/HSPC cellular communication in the bone marrow, both under healthy and malignant conditions (6–8). EVs are submicron-sized membrane vesicles released by virtually every cell type (9). These lipidic bilayer structures act as carriers for different types of signals (proteins, miRNAs, lipids…) and their content reflects the physiological state of the donor cells. Exosomes belong to a subtype of signaling EVs, with a diameter of 40–150 nm, and are secreted in the extracellular space both under physiological and pathological conditions (10). Among the 28 niche cell types estimated in the bone marrow (11), many of them have been proposed to release exosomes that control homeostatic HSPC proliferation, self-renewal and differentiation (8, 12–20). Furthermore, each cell type can release a heterogeneous population of exosomes, increasing the niche’s EV complexity (21). During emergency hematopoiesis triggered by acute infection or inflammation, niche cells adapt HSPC self-renewal and multi-lineage differentiation accordingly *via* exosomes (6). However, how niche cells modulate their exosome production in response to external cues in order to provide target HSPCs with the appropriate signaling, remains largely unknown.

*Drosophila* is a well-established model to study hematopoiesis, both under homeostatic and inflammation-like conditions elicited by pathogen attacks (22, 23). The *Drosophila* larval hematopoietic organ, the lymph gland, develops in contact with the vascular system and is composed of lobe pairs flanking the cardiac tube (24–26). In healthy third instar larvae, the anteriormost lobes of the lymph gland are divided into an inner medullary zone (MZ), containing a heterogeneous population of blood cell progenitors, an outer cortical zone (CZ), composed of differentiated myeloid-like cells, the plasmatocytes and crystal cells, and a non-hematopoietic group of specialized cells called the posterior signaling center (PSC) (24). Between the MZ and CZ, an intermediate zone (IZ) has recently been described, through which cells at various stages of maturation transit (27–32). The PSC and adjacent cardiac cells act as a microenvironment/niche to tune the balance between maintenance of lymph gland progenitors and their differentiation into the myeloid-like cells (33–37). Under conditions of immune stress, such as wasp parasitism, the PSC niche is essential to adapt blood cell progenitor homeostasis to the immune challenge, by promoting rapid and massive differentiation of lymph gland progenitors into lamellocytes, a blood cell type dedicated to the neutralization of the wasp egg laid inside the body of the *Drosophila* larva (25, 33, 38–43). Wasp infestation induces an oxidative stress in PSC cells resulting in the activation of two signaling pathways, EGFR and Toll/NF-κB, in the lymph gland (39, 41). These pathways promote lamellocyte differentiation in the lymph gland, and subsequently their release into the circulation after lymph gland rupture, leading to wasp egg encapsulation and neutralization (25, 41, 44, 45). The *Drosophila* EGFR ligand Spitz, produced by PSC cells, triggers non-cell autonomously EGFR signaling in blood cell progenitors. How Toll/NF-κB pathway activation in niche cells regulates lamellocyte differentiation remains unknown.

Although signaling pathways supporting PSC/progenitor communication upon parasitism in the lymph gland are progressively identified, the mechanisms by which this communication occurs remain elusive. Filopodia-like protrusions emanating from PSC cells and extending over 2 to 3 cell progenitor diameters have been described (33, 34, 46). Such structures, however, could hardly account for PSC signaling throughout all of the lymph gland anterior lobes, where distant progenitors are located many cell diameters apart. Here, we provide evidence that the PSC delivers signaling exosomes to communicate with progenitors during wasp infestation. PSC-derived exosomes, among which a subset can transport the EGFR ligand Spitz, propagate throughout the lymph gland in response to elevated ROS levels in the niche, and activate non-cell autonomously EGFR signaling in lymph gland progenitors. This leads to lamellocyte differentiation, lymph gland rupture, and ultimately the success of wasp egg encapsulation. Our data further show that the metalloproteinase Mmp1 produced by the PSC niche provides a supportive microenvironment, allowing exosomes to cross the niche extracellular matrix, thus promoting their spreading into the lymph gland. This work identifies an exosome-mediated cell communication in the lymph gland, between the hematopoietic niche and blood progenitors, that functions in an anti-parasitic defense.

## Materials and methods

### Fly strains

The following strains were used: *w^1118^* (wild type, *WT*), *Antp-Gal4* (34), *pcol-Gal4 (col>)* and *UAS-mCD8-GFP* (33), *UAS-cSpi, gstD-lacZ* and *Vkg::GFP* (41), *UAS-CD63-GFP* (47), *UAS-HRP-CD8* (48); *UAS-VPS4^DN^* (49). *mCherry::Rab5*, a transgene expressing Rab5 under endogenous regulatory elements (50), Ubiquitin-*Rab11-mcherry* (35), *UAS-Mmp1-RNAi* (51), *UAS-Mmp1^E225A^* (52), DoxA3::GFP (v318882; VDRC Stock Center). The strain *UAS-cSpi-GFP* was kindly provided by B.Shilo. Other *Drosophila* lines were obtained from the Bloomington (BL) stock center: *UAS-CD63* (BL82215)*, UAS-CD63-mCherry* (BL91389), *UAS-2XEGFP* (BL6658), *UAS-catalase* (BL24621), *UAS-TIMP* (BL58708), *UAS-Rab27-RNAi* (BL31887), *UAS-Rab27-RNAi#2* (BL50537), *UAS-Mmp1-RNAi#2* (BL31489), *UAS-Mmp1 (BL58701), tub-Gal80^ts^* (BL7019). Controls correspond to Gal4 drivers crossed with *w^1118^*. For all RNAi experiments, *UAS-Dicer2* (BL24650) was introduced and *Drosophila* development proceeded at 22 °C until late L2 stage, before shifting to 29 °C. For Gal80^ts^ temperature shift experiments, larvae were raised at restrictive temperature (18 °C) and switched to permissive temperature (29 °C) at late L2 stage. For all other conditions, *Drosophila* development proceeded at 25 °C.

### Immunostaining

Lymph glands were dissected and processed as previously described (33). To immunodetect surface-exposed CD63 (extracellular CD63, CD63^ext^) or Mmp1 (Mmp1^ext^), a detergent free protocol was used. Briefly, lymph glands were dissected in Schneider’s media (Gibco 21720024), rinsed with PBS 1X, and fixed with 4% PAF/PBS 1X (Sigma-Aldrich) for 30 min at room temperature (RT). After washing in PBS 1X, lymph glands were incubated with primary antibody (1h30 at RT), washed in PBS 1X and stored overnight at -20 °C in methanol. Incubation with fluorescent secondary antibodies was performed as in (33). Primary antibodies used were mouse anti-CD63 (DSHB, H5C6), mouse anti-Ubi (FK2, 1/200), mouse anti-β-Integrin (DSHB, CF.6G11), mouse anti-diphosphorylated Erk kinase (pERK) antibody (1/100, Sigma-Aldrich), chicken anti-βgal (1/1000, Abcam), rabbit anti-Trol (1/1000, gift from S. Baumgartner), rabbit anti-αPS4 (1/200) (33), mouse anti-Coracle (DSHB, C566.9 and C615.16), mouse anti-Mmp1 (DSHB, 3A6B4 and 14A3D2: a cocktail of two anti-Mmp1 monoclonal antibodies raised against the catalytic and C-terminal domains), mouse anti-Mmp2 (1/200, gift from K. Broadie). Secondary antibodies were Alexa Fluor-488 and -555 conjugated antibodies (1/1000, Molecular Probes) and goat anti-Chicken Alexa Fluor-555 (1/800, Molecular Probes). Nuclei were labeled with either DAPI (Thermo Fisher Scientific) or TOPRO3 (Thermo Fisher Scientific). Samples were mounted in Vectashield (Vector Laboratories) and kept at 4 °C until imaging. Fluorescent images were acquired using a Leica SP8 confocal microscope, or for super-resolution imaging a Zeiss LSM 880 confocal microscope at 63× objective.

### Wasp parasitism, lymph gland rupture and wasp egg encapsulation

In all experiments, genotypes were analyzed in parallel. Late second instar *Drosophila* larvae raised at 22 °C were subjected to parasitism for 1h by *Leptopilina boulardi* (G486 avirulent strain) (53). For RNAi experiments, larvae were shifted to 29 °C 12h before wasp infestation. Lymph gland dispersal and wasp egg hatching were examined in non-fixed parasitized larvae. For lymph gland dispersal, lymph gland anterior lobe integrity was analyzed 10 to 13h post parasitism. Lymph glands were classified into three groups: disrupted when anterior lobes were absent or rudimentary, disrupting when some cells had escaped the anterior lobes, or intact when the anterior lobe border was regular. The score (%) of lymph gland disruption was calculated by dividing the number of lymph glands in each group by the total number of infected larvae. Wasp egg encapsulation was analyzed 48h post parasitism, and the number of *Drosophila* larvae containing melanized/unhatched wasp eggs or living hatched wasp larvae was counted. The % of wasp egg encapsulation was calculated by scoring the number of melanized (black) wasp eggs inside the body of the dissected fly larvae, divided by the total number of infected fly larvae. Graphs and statistical analyses were performed using GraphPad Prism 5 software. *p*-values were calculated with Pearson’s Chi-squared test. Each experiment was repeated independently at least three times, and quantification represents the mean of at least three independent experiments.

### Visualization and quantification of fluorescent EVs

3D confocal images of lymph glands were acquired using either a SP8 confocal microscope at 40X and 63X objectives or, for super-resolution, a Zeiss LSM 880 confocal microscope at 63X objective. In each experiment, identical acquisition settings were used for all genotypes analyzed in parallel. ROIs corresponding to lymph gland anterior lobes (visualized by DAPI staining) and PSC coordinates for calculating EV distances were defined manually. Anterior lobe volume (in µm^3^), the number of fluorescent punctae and their distribution in each lymph gland anterior lobe were measured automatically using Fiji distribution (imageJ 2.16/1.54p) and 3D maxima finder function (3D ImageJ Suite) (54). EV index corresponds to the number of mCD8-GFP or CD63-GFP positive punctae per lymph gland anterior lobe volume. To quantify CD63^ext^ intensity per PSC, we imaged extracellular CD63 (CD63^ext^) by using super-resolution confocal microscopy and measured total intensity of CD63^ext^ for each z-stack representing a single PSC, using Bitplane Imaris software (8.4).

### Electron microscopy

Inverted larvae were fixed with 2.5% glutaraldehyde and 2% paraformaldehyde in cacodylate buffer (0.1 M, pH 7.2, EMS, Hatfield, PA) 2h at room temperature, and HRP activity was revealed by incubation in DAB (5 mg/ml) + 0.003% H_2_O_2_ for 30 min. Larvae were post-fixed with 1%OsO4 for 1h, lymph glands were included in 1% low melting agarose before treatment for 1h with 1% aqueous uranyl acetate. Then samples were dehydrated in a graded ethanol series and embedded in Epon (EMBed-812, EMS). After 48h of polymerization at 60 °C, ultrathin sections (80 nm thick) were mounted on Formvar-coated copper slot grids. Finally, sections were stained with Uranyless and lead citrate (em-grade.com). Grids were examined with a TEM (Jeol JEM-1400, JEOL Inc, Peabody, MA, USA) at 80 kV. Images were acquired using a digital camera (Gatan Orius, Gatan Inc, Pleasanton, CA, USA).

### Quantification of lamellocyte and p-ERK intensities

Lamellocytes were visualized either by expression of DoxA3::GFP or by immunostaining with an antibody against β-Integrin. *p-ERK* expression was visualized by immunodetection with an anti-*p-ERK* antibody. Optimized z-stacks of lymph glands were made using the SP8 confocal microscope. Nuclei were labeled by DAPI to measure the volume of lymph gland anterior lobes. The volumes (in µm^3^) of β-integrin, DoxA3::GFP and *p-ERK* in each lymph gland anterior lobe were measured using Fiji software and 3DSuite plugin (54). Lamellocyte index corresponds to β-integrin or DoxA3::GFP volume/anterior lobe volume, and *p-ERK* index corresponds to *p-ERK* volume/anterior lobe volume. At least 12 anterior lobes per genotype were scored, and experiments were reproduced at least three times. Graphs and statistical analyses t-test (Mann–Whitney nonparametric test) were performed using GraphPad Prism 5 software.

## Results

### The PSC niche releases exosomes

**Figure 1:**
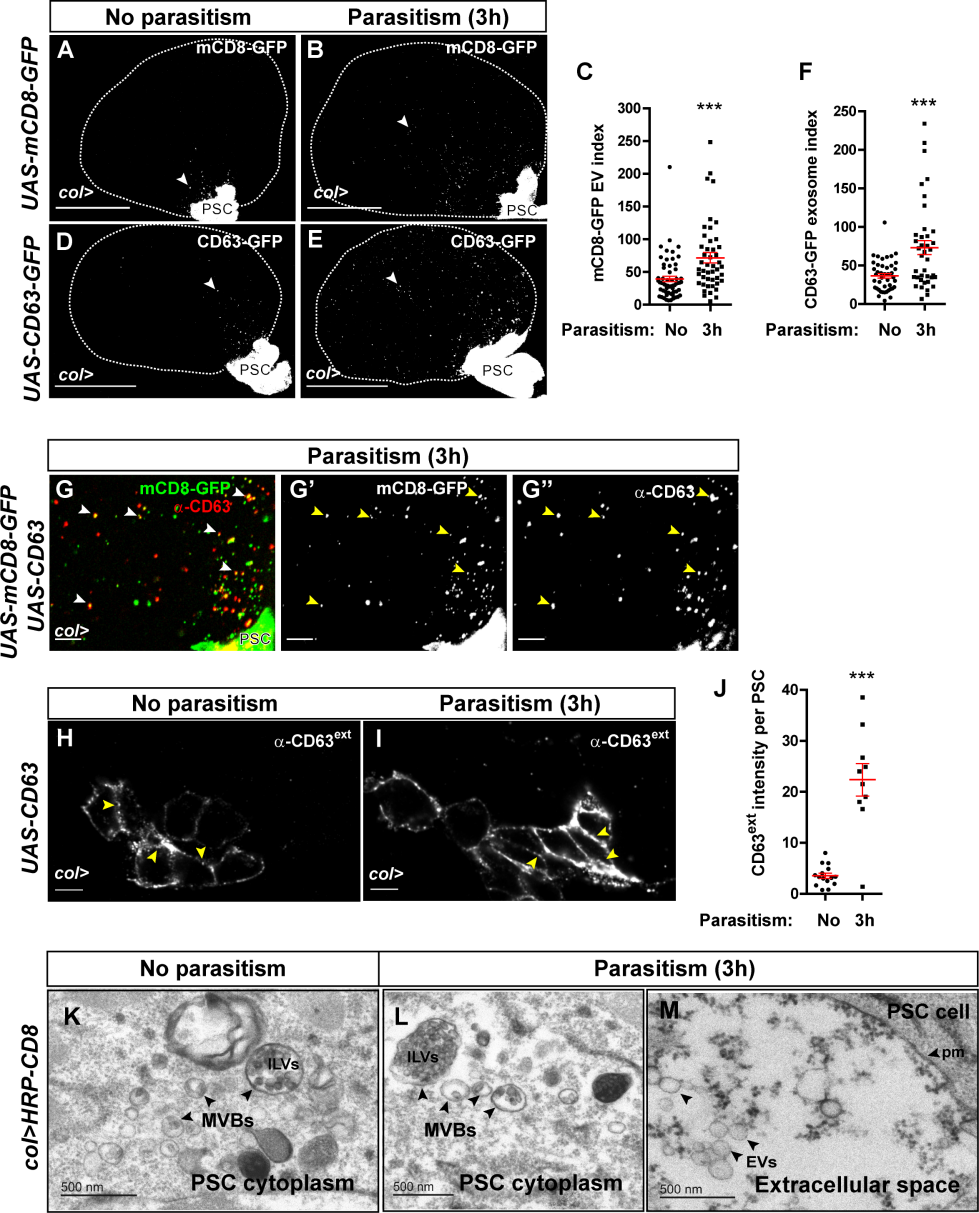
Parasitism stimulates exosome release by the PSC niche. **(A, B, D, E)** Representative confocal image (z-projection) of GFP fluorescent punctae in lymph gland anterior lobe of late second instar (LL2) larvae expressing in PSC cells either a membrane-tethered GFP (*col>mCD8-GFP*) **(A, B)** or the exosome marker CD63-GFP (*col>CD63-GFP*) **(D, E)** in the absence of parasitism **(A, D)** and 3h post-parasitism **(B, E)**. Lymph gland anterior lobes are delineated by broken white lines and arrowheads indicate the GFP fluorescent punctae. **(C, F)** EV indexes (number of GFP positive punctae per anterior lobe volume) in *col>mCD8-GFP* **(C)** and *col>CD63-GFP* lymph glands **(F)** in non-parasitized conditions and 3h post-parasitism. For all vesicle indexes and in all figures: each point corresponds to a single lymph gland anterior lobe, error bars represent SEM, t-test (Mann-Whitney nonparametric test): ***p<0.001. The experiment was repeated independently at least three times with similar results and one is shown. **(G, G’, G’’)** Close up view of anterior lobe in *col>mCD8-GFP>CD63* lymph gland immuno-stained for CD63. mCD8-GFP (green in **G**, white in **G’**) and CD63 (red in **G**, white in **G”**). Only one fraction of EVs released by PSC cells display both fluorescence [white arrowheads in **G**, yellow in **G’** and **G”**). **(H, I)** Super-resolution confocal imaging (single z section) of extracellular CD63 (CD63^ext^, white) at the surface of PSC cells in *col>CD63* lymph glands in normal (non-parasitized) conditions **(H)** and 3h post-parasitism **(I)**. CD63^ext^ is revealed by anti-CD63 immunostaining under non-permeabilizing conditions (see Material and Methods). Yellow arrows: CD63^ext^-postive exosomes at PSC cell junctions. **(J)** Quantification of CD63^ext^ intensity per PSC in *col>CD63* lymph glands. Error bars represent SEM, ***p<0.001 t-test (Mann-Whitney nonparametric test). The experiment was repeated at least three times with similar results and one is shown. **(K–M)** Electron microscopy imaging of PSC cells in *col>HRP-CD8* lymph glands. Arrowheads in K and L indicate multi-vesicular bodies (MVBs), filled with intra-luminal vesicles (ILVs), in the cytoplasm of PSC cells under normal conditions **(K)** and 3h post-parasitism **(L)**. **(M)** EVs (arrowheads) of diameters ranging from about 50 to 170 nm in the extracellular space close to a PSC cell membrane (pm). Note the size heterogeneity of EVs. Scale bars: **(A, B, D, E)** 40 μm, **(G-G”,H, I)** 5 μm, **(K–M)** 500nm.

Expression of a membrane-bound form of GFP (*UAS-mCD8-GFP*) under the control of two PSC-specific-drivers (*col-Gal4* and *antp-Gal4*) revealed fluorescent GFP punctae dispersed throughout the anterior lobes of the lymph gland in control (non-parasitized) larvae (**Figure 1A**; **Supplementary Figure 1A**). Since these drivers are expressed exclusively in PSC cells and not in other cells of the anterior lobes (33, 34), we concluded that the GFP punctae are produced and released by the PSC niche. Previous work established that the PSC controls the cellular immune response to wasp parasitism (33, 38–42), which prompted us to examine mCD8-GFP fluorescence in lymph glands under parasitism. We observed that 3h post-parasitism the number of mCD8-GFP punctae increased significantly in lymph gland anterior lobes of parasitized larvae compared to non-parasitized larvae (**Figures 1A–C**; **Supplementary Figures 1A–C**). In contrast, they were barely detectable 3h post-parasitism in lymph gland anterior lobes when we expressed a cytosolic GFP in PSC cells (*col>eGFP)* (**Supplementary Figures 1D-D**’’). This indicates that the fluorescent punctae enclose mCD8-GFP-marked membrane derived from PSC cells, which is reminiscent of extracellular vesicles (EVs). Altogether, these data suggest that the PSC delivers EVs to lymph gland anterior lobes, and that wasp parasitism is a regulator of this release.

To further characterize the PSC-derived EVs, we expressed in PSC cells the fluorescent tetraspanin CD63-GFP, a mammalian exosome marker used previously to label fly exosomes (47, 55–57). Whereas a few CD63-GFP fluorescent punctae were detected in lymph gland anterior lobes of non-parasitized *col>CD63-GFP* larvae, a high number was found upon parasitism (**Figures 1D–F**), indicating that PSC cells release exosomes in response to parasitism. Yet, anti-CD63 immunostaining revealed that CD63 exosomes correspond to only a sub-population of PSC-derived mCD8-GFP-positive vesicles (**Figures 1G–G**’’), indicating that PSC cells release a heterogeneous population of EVs. CD63 exosomes are also detected by anti-CD63 immunostaining in non-permeabilized lymph glands using an antibody that detects surface-exposed CD63 (CD63^ext^), revealing that CD63 exosomes localize in the extracellular space of anterior lobes (arrowheads in **Supplementary Figures 1E–E**”). To assess the release of CD63 exosomes at the source, we monitored CD63^ext^ at the surface of PSC cells using super-resolution confocal imaging. A fourfold increase of CD63^ext^ intensity, corresponding to either isolated or clustered CD63^ext^-marked exosomes, was detected at the surface of PSC cells 3h post-parasitism (arrowheads in **Figures 1H–J**).

To analyze the kinetics of PSC-derived exosome release following parasitism, we performed a quantitative time-course analysis. We found that the number of CD63 exosomes transiently increased in lymph gland anterior lobes during the first 4h post-parasitism, then receded back to non-parasitized levels by 4-8h post-parasitism (**Supplementary Figure 1F**). In addition, we measured the distance travelled by exosomes from their source (**Supplementary Figure 1G**). An increased proportion (10 to 20%) of CD63 exosomes was found at distant positions between 1 to 4h post-parasitism. Altogether, these data show that parasitism transiently stimulates both exosome release and their spreading in lymph gland anterior lobes.

To visualize exosome biogenesis in PSC cells, we performed electron microscopy on lymph glands. PSC cells were identified by targeted expression of a membrane-bound form of horseradish peroxidase (HRP) (*col>UAS-HRP-CD8*) that produces an electron-dense precipitate at PSC cell membranes (58, 59) (**Supplementary Figure 1H**). Both in mammals and in *Drosophila*, exosomes form as intra-luminal vesicles (ILVs) inside morphologically distinctive endosomes, called multi-vesicular bodies (MVBs) (9, 60). Following maturation, MVBs either fuse with lysosomes to be degraded, or fuse with the plasma membrane for exosome release. EM analysis revealed a heterogeneous population of MVBs, both in size and in ILV number, in the cytoplasm of PSC cells under normal and parasitized conditions, suggesting ongoing MVB maturation in both conditions (**Figures 1K, L**). In addition, EV-like structures of various diameters were present in the extracellular space in close vicinity to the plasma membrane of PSC cells 3 h post-parasitism (**Figure 1M**), consistent with the fluorescent detection of a heterogeneous population of EVs released by PSC cells in response to parasitism (**Figures 1G–G**”). Altogether, these results reveal that the PSC niche is a source of exosomes and that wasp parasitism stimulates their release and their spreading into lymph gland anterior lobes.

### Niche-derived exosomes regulate the lymph gland response to parasitism

**Figure 2:**
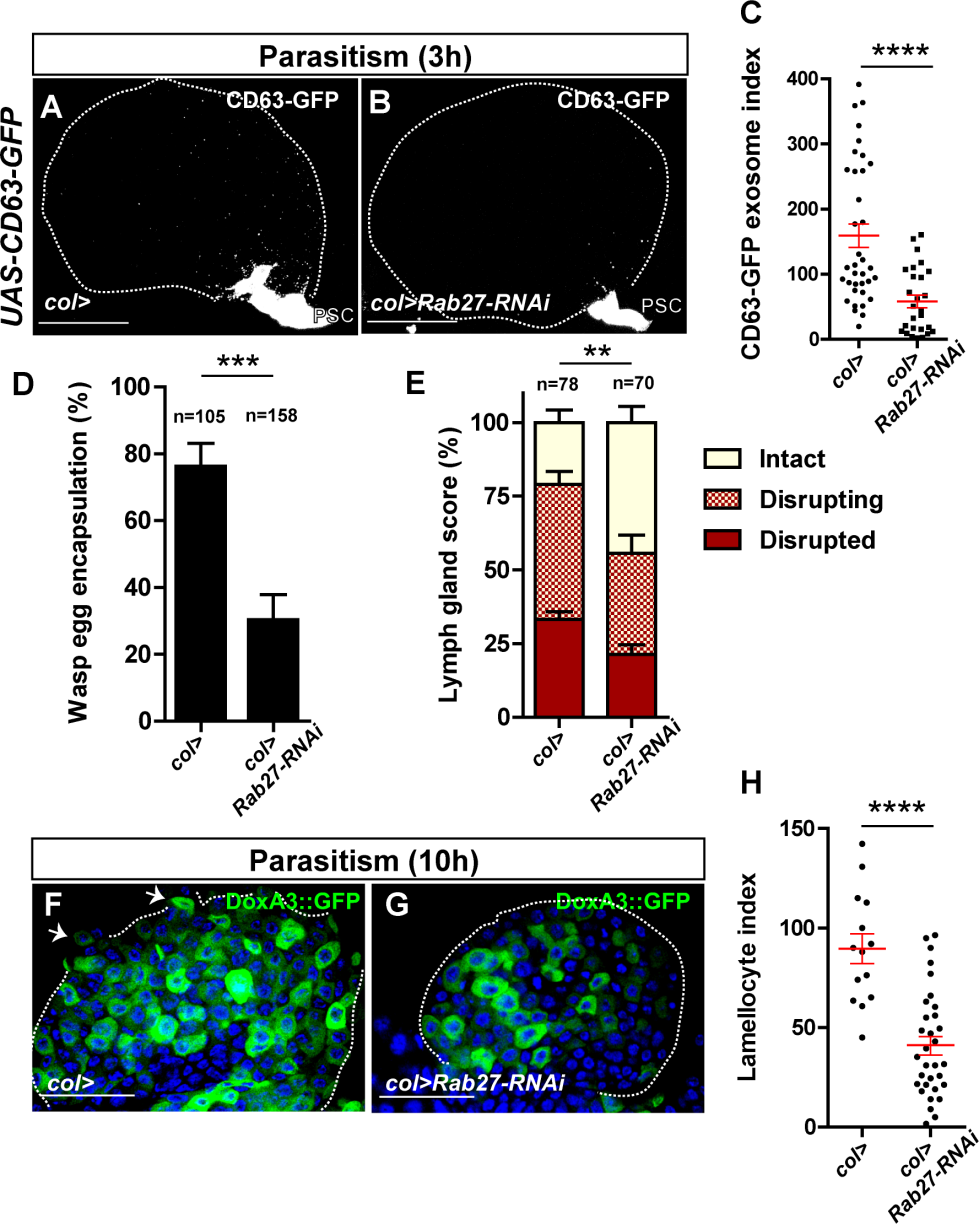
Exosomes released by the PSC niche mediate the *Drosophila* anti-parasitic response. **(A, B)** Confocal image (z-projection) of CD63-positive exosomes in lymph gland anterior lobe of control *col>CD63-GFP* **(A)** and *col>CD63-GFP>Rab27-RNAi* **(B)** larvae. **(C)** CD63-GFP exosomes index 3h post-parasitism in lymph gland anterior lobes. Error bars represent SEM, t-test (Mann-Whitney nonparametric test): ****p<0.0001. **(D)** Quantification (%) of wasp egg encapsulation. Box plots represent the mean of at least three biological replicates. In this and subsequent similar analyses, numbers of (parasitized) larvae analyzed (n) per genotype are indicated for each box plot. Error bars correspond to SEM, ***p<0.001 (Pearson’s Chi-squared test). **(E)** Quantification (%) of lymph gland disruption. Box plots represent the mean of at least three biological replicates. In this and subsequent similar analyses, numbers of larvae analyzed are indicated on histograms. Error bars correspond to SEM, **p<0.01 (Pearson’s Chi-squared test). **(F–G)** Representative confocal image of lamellocyte staining (DoxA3::GFP, green) 10h post-parasitism in control lymph glands (*col>)* and when *Rab27* is knocked down in PSC cells (*col>Rab27-RNAi*). Arrows in F indicate lymph gland rupture at the onset of lamellocyte dispersal. **(H)** Lamellocyte index in lymph gland anterior lobes of the indicated genotypes. ****p<0.0001 (t-test; Mann-Whitney nonparametric test). All experiments were repeated independently at least three times with similar results. Scale bars: 40 μm.

Multiple studies have proposed that EVs play a critical role in facilitating adaptation to cellular stress (6, 8, 16, 62–64). During wasp parasitism, lymph gland homeostasis switches from homeostatic to stress-induced hematopoiesis. The PSC plays a critical function in this switch by instructing the differentiation of lamellocytes that are required to block wasp egg hatching (38, 40, 42). Thus, we asked whether the PSC function relies on EV secretion by knocking-down in PSC cells cellular components implicated in exosome biogenesis and secretion. Rab27 promotes exosome secretion by targeting MVB to the plasma membrane for fusion, while the ATPase Vps4 regulates ILV biogenesis inside the lumen of MVBs (60, 65–67). Previous studies have indicated that knocking down Rab27 or expressing a dominant negative form of Vps4 (VPS4^DN^) impairs exosome release in *Drosophila* cultured cells and tissues (49, 55, 60, 68, 69). We found that knocking-down Rab27 in PSC cells (*col>Rab27-KD*), using two different *UAS-RNAi* lines, significantly reduced the release of both CD63 exosomes and mCD8-GFP EVs 3h post-parasitism compared to controls (**Figures 2A–C**; **Supplementary Figure 2A**). Similar results were obtained by expressing VPS4^DN^ in PSC cells (*Gal80^ts^;col>VPS4^DN^*) (**Supplementary Figures 2B–D***)*. Note that, as previously reported (70), knocking-down Vps4 also causes intracellular accumulation of ubiquitinated proteins as a result of defective endosomal-lysosomal trafficking (**Supplementary Figures 2E–F'**), which may interfere with our observations. In contrast, knocking-down Rab27 does not affect this endosomal trafficking (**Supplementary Figures 2G–H'**) (60). Therefore, *Rab27-KD* conditions were used in subsequent experiments to specifically disrupt exosome release from PSC cells following parasitism.

Following parasitic infestation, wasp egg encapsulation and lymph gland rupture reliably reflect the success of the *Drosophila* cellular immune response (41, 71). We therefore investigated whether exosome release from PSC cells plays a role to fight wasp parasitism. Wasp egg encapsulation was impaired in *col>Rab27-KD* larvae compared to control (**Figure 2D**). Similar results were obtained using a distinct *UAS-RNAi* line and another PSC driver (*Antp>*) (**Supplementary Figure 2I**). We then scored the percentage of intact, disrupting and dispersed lymph glands (see material and methods). While 75% of lymph glands showed disrupting and disrupted lobes in control larvae 12h post parasitism, only 55% of them showed a response in parasitized *col>Rab27-KD* larvae (**Figure 2E**), which indicates that interfering with exosome release impairs lymph gland rupture. Accordingly, fewer lamellocytes labeled by DoxA3 or *β-integrin* (72, 73) were observed 10h post-parasitism in col>Rab27-KD lymph glands compared to control (**Figures 2F–H** and **Supplementary Figures 2J–L**). This shows that lamellocyte differentiation, while still occurring in *col>Rab27-KD* lymph glands, is reduced compared to the control. Altogether, our data establish that exosome delivery by PSC cells is required for appropriate differentiation of lymph gland lamellocytes and on time dispersal from the organ, enabling successful wasp egg encapsulation.

### Niche-derived exosomes mediate EGFR signaling in the lymph gland

**Figure 3:**
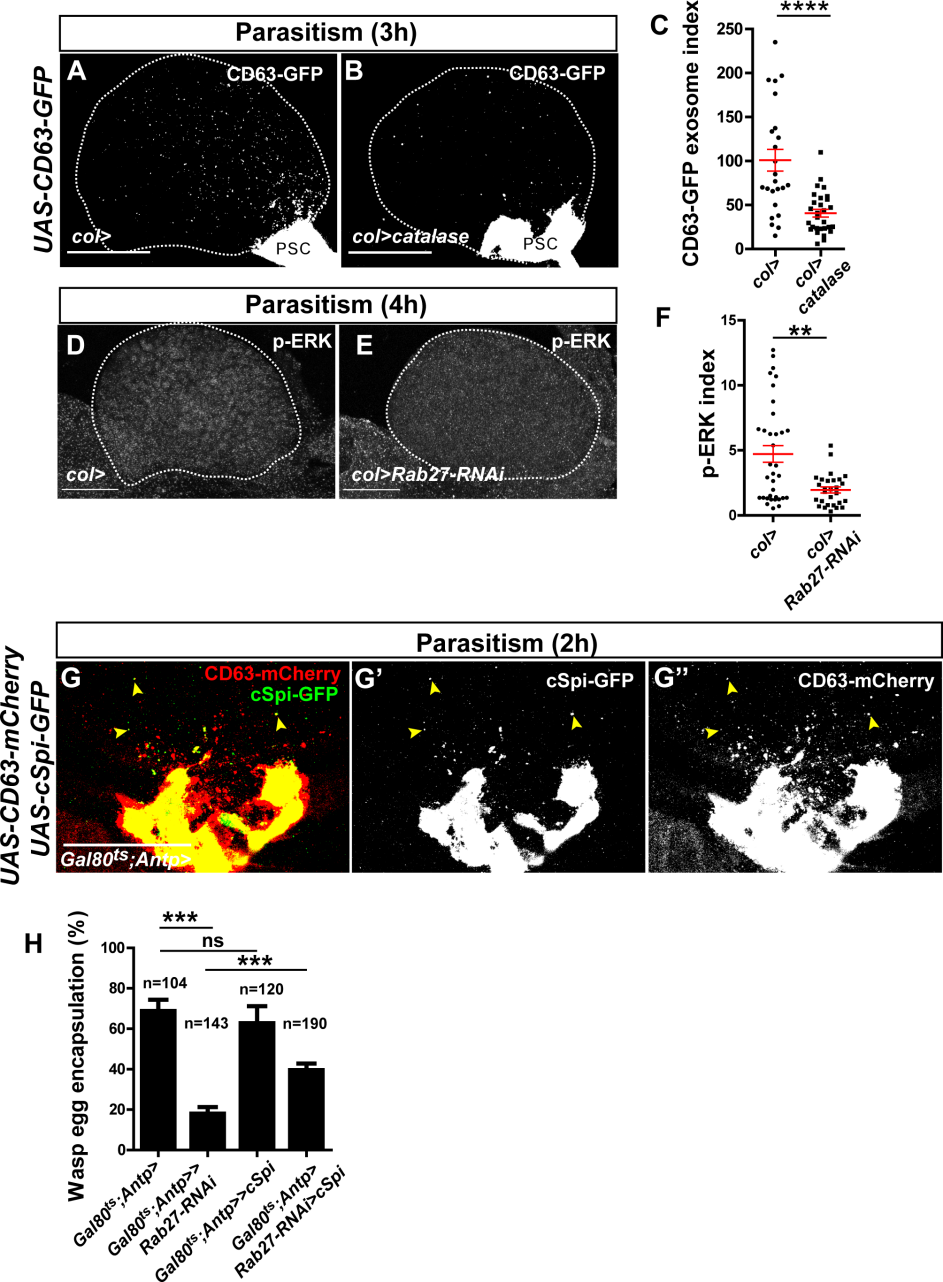
Oxidative stress in the niche stimulates exosome release by PSC cells which in turn activates EGFR signaling in lymph gland progenitors. **(A, B)** Confocal image (z-projection) of CD63-GFP exosomes in lymph gland anterior lobe of control *col>CD63-GFP* **(A)** and *col>CD63-GFP>catalase* **(B)** larvae 3h post-parasitism. **(C)** CD63-GFP exosome index 3h post-parasitism in lymph gland anterior lobes. Error bars represent SEM, t-test (Mann-Whitney nonparametric test): ****p<0.0001. **(D–E)** Representative confocal image of p-ERK (white) immunostaining in control lymph gland (*col>*) **(D)** and when *Rab27* is down-regulated in PSC cells (*col>Rab27-RNAi*) **(E)** 4h post-parasitism. **(F)** p-ERK index in lymph gland anterior lobes. Error bars represent SEM. **p<0.01 (t-test, Mann-Whitney nonparametric test). **(G-G”)** Close up view of the PSC in *Gal80^ts^;Antp>CD63-mCherry>cSpitz-GFP* lymph gland 2h post-parasitism. Co-localization of cSpitz-GFP (green in **G**, white in **G’**) and CD63-mCherry exosomes (red in **G**, white in **G”**) are indicated by yellow arrowheads. **(H)** Quantification (%) of wasp egg encapsulation. Box plots represent the mean of at least three biological replicates. Error bars correspond to SEM, ***p<0.001 and ns (not significant) (Pearson’s Chi-squared test). Scale bars: 40 μm.

Since exosomes are required for PSC function in response to parasitism, we further investigated the underlying molecular mechanism. An early and critical step induced by parasitism is an increase of ROS levels in PSC cells (39). Interestingly, oxidative-stress conditions have been reported to stimulate exosome production and release both in flies and in mammals (64, 74–76). These data prompted us to investigate the relationship between the oxidative stress in the PSC niche following parasitism and its exosome release. We found that ROS levels in PSC cells, visualized by the expression of the *gstD-lacZ* transgene, increased as soon as 2h after wasp parasitism (**Supplementary Figures 3A–B**), which correlates with the timing of exosome release by PSC cells (**Supplementary Figure 1F**). Then, we tested the effect on EV release of reducing PSC ROS by expressing an anti-oxidant intracellular catalase (*col>UAS-catalase*) (41). Levels of CD63-positive exosomes released by PSC cells were strongly decreased in lymph gland anterior lobes of *col>UAS-catalase* larvae 3h post parasitism, compared to the control (**Figures 3A–C**). In addition, lower levels of CD63^ext^ intensity were detected at the surface of PSC cells, indicating that CD63^ext^-marked exosomes diminished significantly (**Supplementary Figures 3C–E**). We concluded that the oxidative stress induced by parasitism in PSC cells increases exosome release from the PSC niche.

Elevated ROS lead to the secretion of the cleaved, active EGF ligand Spitz (cSpi) by PSC cells that activates EGFR signaling in lymph gland progenitors, promoting lamellocyte differentiation and lymph gland rupture (39, 41). Since PSC-derived exosomes disperse throughout lymph gland anterior lobes (**Figure 1**), we asked whether they are the effectors of long-range EGFR activation in lymph gland progenitors. We knocked down *Rab27* in PSC cells to hinder exosome release and looked at phosphorylated ERK (p-ERK), a readout for EGFR activation 4-5h post-parasitism (39, 41). The reduction of exosome release uniformly reduced the p-ERK signal post parasitism in lymph gland anterior lobes compared to the control (**Figures 3D–F**), indicating that PSC-derived exosomes are required for EGFR signaling throughout lymph gland progenitors. Since exosome signaling in recipient cells may occur via endocytosis (61), we performed co-detection of CD63-positive exosomes and endogenous markers for early (Rab5) (**Supplementary Figures 3F–F**”) and recycling (Rab11) (**Supplementary Figures 3G–G**”) endosomes. We observed that some CD63 exosomes colocalize with these markers in lymph gland progenitors, suggesting that exosomes derived from the PSC can be internalized. Altogether, these data strongly suggest that niche-released exosomes mediate cell communication in the lymph gland in response to parasitism and promote long-range EGFR pathway activation throughout lymph gland anterior lobes.

Since exosomes transport cargoes to recipient cells (61), and since PSC-derived exosomes activate EGFR signaling, we reasoned that cSpitz, the active EGF ligand produced by PSC cells (39), could be carried by PSC-derived EVs. To test this hypothesis, and because of unsuccessful Spitz immuno-detection in the lymph gland, we drove the expression of cSpitz in PSC cells (*Gal80^ts^;Antp>cSpi-GFP*). We detected punctate cSpi-GFP fluorescence released by PSC cells 2h post-parasitism (**Figures 3G–G**”). Co-detection of CD63 exosomes revealed that only a subset of cSpi-GFP punctae was labeled with CD63, suggesting that cSpitz spreads throughout the lymph gland via both CD63+ and CD63− EVs. Next, we wondered whether wasp egg encapsulation defects observed under conditions of low CD63 EV release in *col>UAS-Rab27-KD* larvae (**Figures 2A–D**) might result from insufficient Spitz supply to the progenitors. Co-expressing cSpitz in PSC cells in parallel to knocking-down *Rab27* (*Gal80^ts^;Antp>UAS-Rab27-RNAi>cSpi*) significantly improved wasp egg encapsulation compared to *col>Rab27-KD* alone (**Figure 3H**), suggesting that the immune deficiency can be partially overcome by increasing Spitz levels released by the PSC. Altogether, these results strongly suggest that, following parasitism, Spitz is transported by PSC-derived EVs, including CD63 exosomes, to activate EGFR signaling in lymph gland progenitors. Hence, exosomes constitute a novel mechanism of cell communication between the PSC niche and hematopoietic progenitors in the *Drosophila* lymph gland.

### TIMP inhibits exosome spreading from the niche

**Figure 4:**
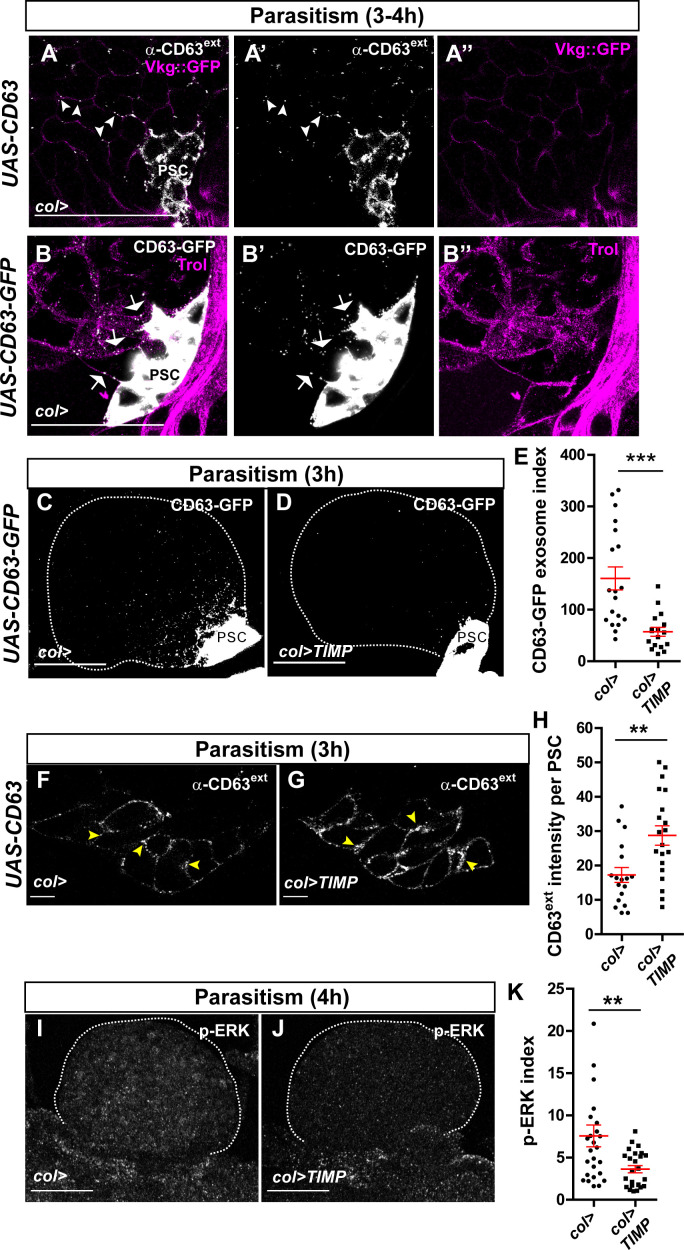
Exosome spreading from the PSC niche requires a protein activity sensitive to TIMP. **(A-A”)** Localization of CD63-positive exosomes (white in A,A’) in the ECM (Vkg::GFP, magenta in **A, A”**) of lymph gland anterior lobe in *col>CD63* larvae immunostained for extracellular CD63 (CD63^ext^) 3-4h post-parasitism. White arrowheads in A and A’ indicate CD63 exosomes localized on the ECM network surrounding progenitor clusters. **(B)** Super-resolution confocal imaging (z-projection) of CD63-GFP exosomes (white in **B, B’**) in the ECM (Trol, magenta in **B, B’**) in *col>CD63-GFP* lymph gland 3-4h post-parasitism. Exosomes (white arrows in **B, B’**) decorate the meshwork of ECM adjacent to PSC cells. **(C, D)** Confocal image (z-projection) of CD63 exosomes in lymph gland anterior lobe of *col>CD63-GFP* (control) **(C)** and *col>CD63-GFP>TIMP* **(D)** larvae 3h post-parasitism. **(E)** CD63-GFP exosome index 3h post-parasitism in lymph gland anterior lobes. Error bars represent SEM, t-test (Mann-Whitney nonparametric test): ***p<0.001. **(F, G)** Super-resolution confocal imaging (single z section) of extracellular CD63 (CD63^ext^, white) at the surface of PSC cells in *col>CD63* and *col>CD63>TIMP* lymph glands 3h post-parasitism. Yellow arrowheads: exosomes at PSC cell junctions. **(H)** Quantification of CD63^ext^ intensity per PSC. Error bars represent SEM, **p<0.01 t-test (Mann-Whitney nonparametric test). **(I, J)** Representative confocal image of p-ERK (white) immunostaining in lymph gland anterior lobe of control (*col>*) **(I)** and *col>TIMP* **(J)** larvae 4h post-parasitism. **(K)** *p-ERK* index in lymph gland anterior lobes. Error bars represent SEM, **p<0.01 (t-test, Mann-Whitney nonparametric test). Experiments were repeated independently at least three times with similar results and one experiment is shown. Scale bars: **(A-D, I, J)** 40 μm, **(F, G)** 5 μm.

In lymph gland anterior lobes, the ECM is present around progenitor clusters (77). PSC cells are also surrounded by a dense meshwork of extracellular matrix (ECM) (30, 77). Exosome detection in the extracellular space of lymph gland anterior lobes (**Supplementary Figures 1E–E**”) prompted us to examine closely the relationship between PSC-derived exosomes and the ECM. Co-detecting CD63 exosomes with the ECM components Collagen IV (Vkg(Viking)::GFP) or Trol/Perlecan showed that PSC-derived exosomes localize to the matrix surrounding progenitor clusters (**Figures 4A–A**” and arrowheads in 4A,A’) and decorate the ECM meshwork encompassing the PSC and neighboring progenitors (**Figures 4B–B**” and arrows in 4B,B’). These localizations indicate the ingress of PSC-derived exosomes into the ECM embedding PSC cells to reach target progenitors, which raises the question of what controls their travelling across the PSC ECM.

To address this question, we asked whether the ECM surrounding PSC cells is degraded following parasitism to allow exosome spreading from their source. Immunostaining for Vkg::GFP or Trol did not reveal obvious changes of ECM around or in the vicinity of PSC cells 3h post-parasitism, by the time of exosome release in lymph gland anterior lobes (**Supplementary Figures 4A–D'**). Neither did we detect change in the expression of integrin αPS4 (**Supplementary Figures 4E–F'**), an ECM receptor known to maintain cell-ECM adhesion in *Drosophila* tissues (78), or of Coracle (**Supplementary Figures 4G–H'**), a septate junction component required to maintain a permeability barrier around PSC cells (79). Since it has been shown that disruption of this barrier activates the cellular immune response during bacterial infection, our results suggest that distinct regulatory mechanisms control signal propagation from the niche during bacterial infection and parasitic infestation. We thus conclude that exosome dispersion from PSC cells during parasitism does not involve major ECM degradation and/or disruption of the PSC cell barrier. To then investigate whether a modification of the ECM other than degradation could be required for exosome spreading, we overexpressed *Drosophila* Tissue inhibitor of metalloproteinases (TIMP) specifically in PSC cells. TIMP is a secreted protein that blocks the metalloproteinase activity known to regulate ECM turnover/remodeling (80, 81). In *col>TIMP* larvae, the number of PSC-derived exosomes was significantly reduced 3-4h post parasitism compared to control (**Figures 4C–E**). Furthermore, CD63^ext^-marked exosomes detected at the surface of the PSC in *col>TIMP* lymph gland increase compared to the control (**Figures 4F–H**). These data show that TIMP expression does not prevent exosome secretion but rather compromises their spreading from the PSC, suggesting that a metalloproteinase activity is required to allow exosomes to ingress the PSC ECM.

To next address the requirement of exosome spreading for efficient immune response to parasitism, we monitored EGFR signaling in the lymph gland and wasp egg neutralization in *col>TIMP* larvae. A reduction of both p-ERK levels in lymph gland progenitors 4h post-parasitism (**Figures 4I–K**) and wasp egg encapsulation efficiency, compared to controls are observed (**Supplementary Figure 4I**). Altogether, these data indicate that exosome spreading from PSC cells and signaling in lymph gland progenitors requires a metalloproteinase activity in the PSC.

### Mmp1 produced by PSC cells controls exosome spreading from the niche

**Figure 5:**
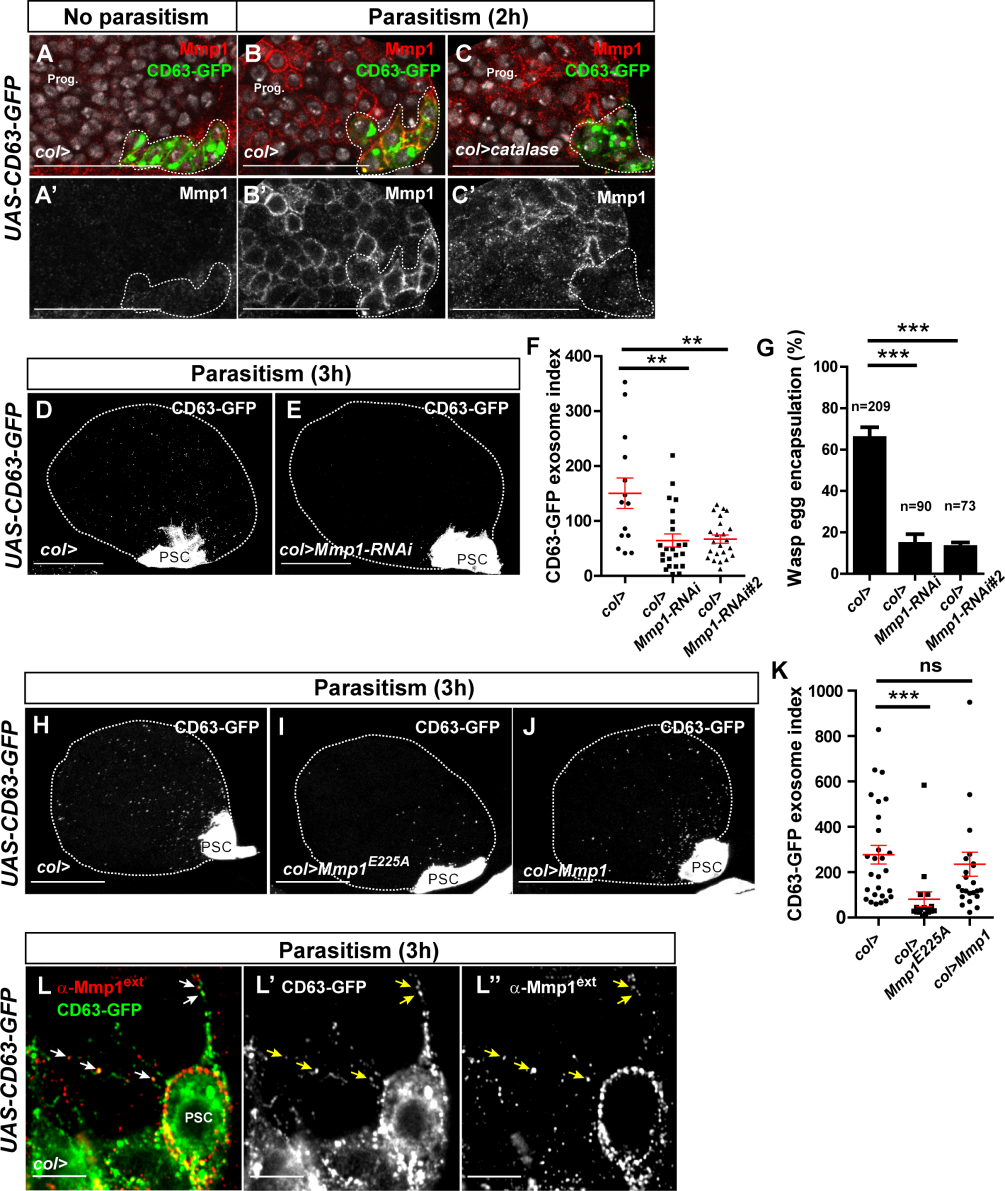
Mmp1 expression in PSC cells is necessary for exosome dispersion from the niche following parasitism. (A–C’) Close up view (single confocal section) of Mmp1 immunostaining (red in **A–C**; white in A’-C’) in lymph gland anterior lobe of control (*col>CD63-GFP*) (A- B’) and when ROS levels are decreased in PSC cells (*col>CD63-GFP>catalase*) (C,C’) without parasitism (A,A’) and 2h post-parasitism (B-C’). Prog, progenitors. The PSC expresses CD63-GFP green in **(A-C)** and is outlined with a dashed line. **(D, E)** Confocal image (z-projection) of CD63-GFP exosomes in lymph gland anterior lobe of control *col>CD63-GFP* **(D)** and *col>CD63-GFP>Mmp1-RNAi* **(E)** larvae 3h post-parasitism. **(F)** CD63-GFP exosome index 3h post-parasitism in lymph gland anterior lobes of the genotypes shown in D and E, and when *Mmp1* is knocked-down in PSC cells using a different *Mmp1-RNAi* line (*col>Mmp1-RNAi#2*). Error bars represent SEM, t-test (Mann-Whitney nonparametric test): **p<0.01. **(G)** Quantification (%) of wasp egg encapsulation. Box plots represent the mean of at least 3 biological replicates. Error bars correspond to SEM, ***p<0.001 (Pearson’s Chi-squared test). **(H–J)** Confocal image (z-projection) of CD63-GFP exosomes in lymph gland anterior lobe of control *col>CD63-GFP* **(H)**, *col>CD63-GFP>Mmp1^E225A^* **(I)**, *col>CD63-GFP>Mmp1* **(J)** larvae 3h post-parasitism. **(K)** CD63-GFP exosome index 3h post-parasitism in lymph gland anterior lobes. Error bars represent SEM, t-test (Mann-Whitney nonparametric test): ***p<0.001 and ns (not significant). (L-L’’) Immuno-detection of extracellular Mmp1 (Mmp1^ext^, red) by using super-resolution confocal microscopy in *col>CD63-GFP* lymph glands 3h post-parasitism. Arrows (white in L, yellow in L’ and L”) indicate Mmp1^ext^-positive punctae that overlap with CD63-positive exosomes. Experiments were repeated independently at least three times with similar results. Scale bars: **(A–E, H, I, J)** 40 μm, (L-L”) 5 μm.

TIMP can modulate diverse proteinase activities and its effect on metalloproteinases (MMPs) is well documented (81). The *Drosophila* genome possesses two MMPs, Mmp1 and Mmp2, playing key roles in ECM remodeling and/or degradation during various developmental processes (81, 82). We addressed Mmp1 and Mmp2 protein expression following parasitism. Mmp2 is expressed in all lymph gland cells, including in PSC cells, without major difference between normal (non-parasitized) and parasitized conditions (**Supplementary Figures 5A–B'**). In contrast, Mmp1 is highly up-regulated 2h post-parasitism in both PSC and progenitor cells, where it accumulates at the cell membrane (**Figures 5A, B'**). In PSC cells, Mmp1 staining co-localizes with discrete CD63 foci (arrowheads in **Supplementary Figures 5C-C**”). Since up-regulation of Mmp1 in PSC cells is concomitant with ROS increase (**Supplementary Figures 3A, B'**), we asked whether Mmp1 up-regulation is controlled by ROS. Decreasing ROS levels in PSC cells (*col>catalase*) prevents Mmp1 up-regulation in these cells 2h post-parasitism, while Mmp1 up-regulation is still detected in progenitors (**Figures 5C, C'**). These results show that Mmp1 is up-regulated in PSC cells in response to ROS increase after parasitism.

Mmp1 up-regulation in PSC cells and the lack of exosome spreading in *col>TIMP* larvae raised the hypothesis that TIMP blocks Mmp1 activity in PSC cells under parasitism. To investigate this possibility, we asked whether Mmp1 is required for exosome spreading from PSC cells by knocking-down its expression using UAS-RNAi lines (*col>Mmp1-KD*). 3h post-parasitism, the numbers of CD63 exosomes detected both in lymph gland anterior lobes (**Figures 5D–F**) and in the ECM in the vicinity of PSC cells (**Supplementary Figures 5D, E**) were significantly reduced in *col>Mmp1-KD* larvae compared to controls. These reductions together with the specific down-regulation of Mmp1 in the PSC of *col>Mmp1-KD* lymph glands, and not in anterior lobe progenitors (**Supplementary Figures 5F–G'**), indicate a cell-autonomous requirement of Mmp1 in PSC cells to control exosome spreading from the PSC niche into the surrounding ECM. Since exosome spreading in the lymph gland is required for a successful anti-parasitic response (**Supplementary Figure 4I**), we anticipated that Mmp1 depletion in PSC cells would impair this response. Accordingly, levels of wasp egg encapsulation were significantly reduced when Mmp1 was knocked-down in PSC cells using different drivers and RNAi lines (**Figure 5G** and **Supplementary Figure 5K**). Altogether, these results show that Mmp1 produced by PSC cells upon parasitism ensures appropriate exosome spreading from the niche into the lymph gland and a robust immune response against wasp parasitism.

To further assess whether the metalloproteinase activity of Mmp1 is involved, we expressed a catalytically-inactive Mmp1 protein in PSC cells (*col>Mmp1^E225A^*), which acts as a dominant negative (83). Numbers of CD63 exosomes were significantly reduced 3h post-parasitism in anterior lobes of *col>Mmp1^E225A^* lymph glands compared to the control, while expressing a wild-type Mmp1 protein (*col>Mmp1*) had no major effect (**Figures 5H–K**). In addition, CD63^ext^-marked exosomes were more abundant at the PSC surface in *col>Mmp1^E225A^* compared to control (**Supplementary Figures 5H–J**). Altogether, these data indicate that the catalytic activity of Mmp1 is required for exosome spreading from the PSC into the surrounding ECM.

The co-localization of Mmp1 with intracellular CD63 foci in PSC cells was intriguing (**Supplementary Figures 5C–C**”) and suggested that Mmp1 localizes to endosomal organelles associated to exosome biogenesis and/or trafficking. To date, the relationship between MMPs and EVs has not been investigated in *Drosophila*. However, exosome-associated MMPs have been reported in mammals, including in cancer contexts, where they contribute to promoting tumor invasiveness through extracellular matrix remodeling (84, 85). We hypothesized that Mmp1 loaded onto exosomes could facilitate exosome spreading from the PSC niche to penetrate the surrounding ECM. To explore Mmp1 localization at the surface of PSC-derived exosomes, we immuno-detected extracellular Mmp1 (Mmp1^ext^) by using super-resolution confocal imaging. Mmp1 is detected at the surface of PSC cells 3h post-parasitism (**Figures 5L–L**”). In the nearby extracellular space, Mmp1^ext^ was detected in numerous punctae, some of them corresponding to CD63 exosomes produced by PSC cells. This data shows that Mmp1 is a surface component of a subset of PSC-derived, CD63-positive exosomes. Altogether, these results strongly suggest that Mmp1 activity associated with PSC-derived exosomes facilitate exosome travelling through the ECM surrounding PSC niche cells.

## Discussion

**Figure 6:**
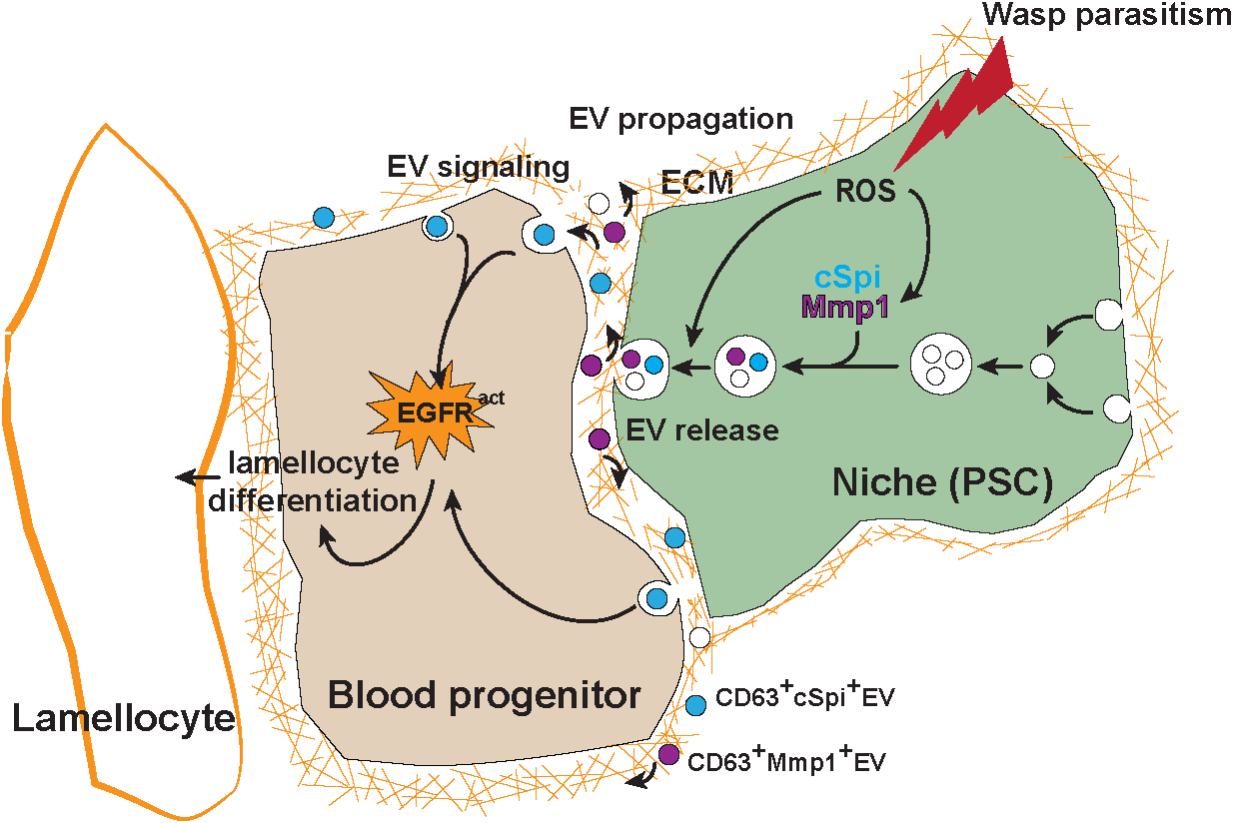
Model of PSC-derived exosome signaling during wasp-induced stress hematopoiesis. Exosomes are produced in PSC cells in MVBs under normal (non-parasitized) conditions. Wasp infestation leads to an increase of ROS in PSC cells that triggers cSpitz production and Mmp1 up-regulation. In addition, ROS stimulates release of exosomes from PSC cells. Exosomes containing Mmp1 and/or cSpitz spread from the niche by traversing the ECM meshwork surrounding PSC cells, possibly thanks to exosome-loading of Mmp1 activity that remodels locally niche ECM structure. cSpitz-containing exosomes activate EGFR signaling throughout blood cell progenitors, ensuring massive lamellocyte differentiation necessary to fight wasp infestation.

In the lymph gland, the hematopoietic niche formed by PSC cells is essential to trigger a cellular immune response dedicated to combat parasitism. The oxidative stress induced in PSC cells by wasp attack leads to EGFR activation in lymph gland blood progenitors, triggering their differentiation into lamellocytes (39, 41). However, how the few PSC cells instruct the entire pool of lymph gland progenitors to become lamellocytes remains largely unknown. Here, we report that in response to oxidative stress induced by parasitism, PSC cells release exosomes that propagate throughout the lymph gland organ (**Figure 6**). Release and propagation of exosomes, among which a subset can carry the Spitz ligand, are required to activate the EGFR pathway in blood progenitors, leading to lymph gland lamellocyte differentiation, on time lymph gland rupture and, ultimately successful wasp egg encapsulation. We further show that exosome spreading from PSC cells into the surrounding ECM, relies on the matrix remodeling activity of Mmp1 produced by PSC cells and loaded onto PSC-derived exosomes in response to wasp parasitism. Hence, we establish that exosome-mediated cell communication in the *Drosophila* lymph gland is essential for the hematopoietic niche control of blood progenitor differentiation in response to a parasitic attack.

In this study, PSC-derived EVs were identified using the mammalian markers CD8 and CD63. Partial colocalization between these markers indicates that PSC cells release a heterogeneous population of EVs in response to parasitic challenge. This heterogeneity is further confirmed by the variable sizes of EVs observed *via* both fluorescence and electron microscopy. Furthermore, the partial defect in wasp egg encapsulation observed upon Rab27 inhibition (**Figures 2**; **Supplementary Figure 2**) may reflect a functional contribution of EV subtypes produced independently of Rab27 to the response to parasitism. It should be noted that filopodia extend from PSC cells (33, 34, 46) and these membrane extensions have been shown to serve as platforms for EV release, independent of the conventional Rab27-dependent EV release *via* MVBs, giving rise to larger EV subtypes known as micro vesicles or apoptotic bodies (61, 86). Thus, it is possible that filopodia-derived EVs contribute to the diversity of PSC-derived EVs that participates in the immune response. Beyond their heterogeneity and the diversity of the mechanisms involved in their biogenesis, the functional properties of distinct EV populations produced by PSC cells, and their potential specialization in transmitting various niche signals, represent fundamental yet challenging questions to address *in vivo.*

How ROS levels control exosome secretion by the PSC niche remains unknown. Exosome release by cells is highly regulated at multiple steps, from ILV biogenesis in MVBs to MVB trafficking towards the plasma membrane for fusion and secretion (9, 87). In *Drosophila* and mammals, exposure to oxidative stress increases both exosome biogenesis and secretion (63, 64, 74–76). Possible mechanisms include elevation of cytosolic calcium, alteration of MVB/lysosomal membrane fusion, modification of lipid membrane levels, and actin cytoskeleton remodeling (74–76, 88). How ROS levels regulate Mmp1 expression in PSC cells also remains to be clarified. In *Drosophila* tissues, as in zebrafish and mammalian cell culture, the JNK pathway controls MMP expression in response to inflammatory stress (51, 89–93). Since JNK signaling is a known effector of ROS in *Drosophila* (94), one possibility is that ROS up-regulate Mmp1 in PSC cells *via* JNK signaling. Thus, additional investigation is required to identify what PSC intracellular signaling downstream of ROS up-regulates both exosome secretion and Mmp1 expression after parasitism.

Our work reveals that niche Mmp1 up-regulation is essential for exosome-mediated cell communication in lymph glands after parasitism. We found that Mmp1 allows exosomes to penetrate the ECM surrounding PSC cells for subsequent propagation throughout the lymph gland. Both TIMP expression and knock-down of Mmp1 activity in PSC niche cells cause exosome stacking at the surface of niche cells, which in turn prevents the niche from triggering the anti-parasitic response. Although we do not fully understand how Mmp1 controls exosome spreading from the niche, our data indicate that Mmp1 concentrates in PSC cells within CD63-positive structures and localizes onto CD63 exosomes released by PSC cells. These data suggest that at least a fraction of Mmp1 associates with exosomes before their secretion and travels with them after release. Interestingly, Mmp1 can be secreted or retained at the cell membrane through a glycophosphatidylinositol (GPI) membrane anchor (52) that could potentially tether Mmp1 to the exosome membrane. Hence, exosome-loading of Mmp1 could promote exosome spreading *in situ* by remodeling locally the ECM around EVs rather than causing general ECM degradation, consistent with no reduction in the niche’s ECM after parasitism (**Supplementary Figure 4**). Notably, in the developing *Drosophila* egg chamber, Mmp1 causes only subtle changes in ECM texture by altering collagen fibril density and length (95). Slight modifications in texture can impact ECM mechanical properties that in turn regulate EV mobility (96–98). The detailed mechanism of how Mmp1 modifies the niche microenvironment to support exosome invasiveness in response to parasitism requires further investigation.

Wasp-derived EV-like structures called venosomes have been shown to invade the *Drosophila* lymph gland during parasitization by the wasp *Leptopilina heterotoma* (99). Venosomes are co-injected during egg deposition into the *Drosophila* circulatory system, where they overcome *Drosophila* defense by killing both circulating hemocytes and lymph gland cells (100). The mechanism by which venosomes enter the lymph gland is unknown, but they are detected in the ECM surrounding PSC cells. Unlike lymph gland cells, PSC cells do not uptake venosomes and, strikingly, PSC ablation prevents venosome invasion of the lymph gland. One possibility could be that venosomes rely on PSC-derived cue(s) for extracellular trafficking into the lymph gland organ.

A main finding of our study is that EGFR signaling via PSC-derived exosomes is a key inducer of lamellocyte differentiation and, thereby the success of wasp egg encapsulation. In *Drosophila*, association of the EGFR ligand Spitz with exosomes has not been reported. Here, we show that cSpi-GFP is detected in both CD63+ and CD63− EVs, indicating that this ligand can be transported by distinct EV subpopulations. Accordingly, simultaneously overexpressing cSpitz and downregulating Rab27 in PSC cells (*Gal80^ts^;Antp>UAS-Rab27-RNAi>cSpi*) (**Figure 3H**) significantly enhances the immune response to parasitism compared to *col>Rab27-KD* alone, suggesting that EVs released independently of Rab27, whether CD63 positive or not, could transport Spitz. Spitz was originally shown to diffuse *in vivo* and act at a distance of several cell diameters (101, 102), whereas other investigations showed that Spitz is tethered to cell membrane by a palmitate group, restricting its diffusion (103–105). Based on their rapid and deep propagation into the lymph gland in response to parasitism, EVs provide a valuable vehicle for the long-range action of Spitz. Consequently, the various modes of extracellular propagation of the Spitz ligand are not mutually exclusive. All forms of Spitz, whether loaded onto different types of EVs or circulating freely in the extracellular space, could collectively contribute to robust EGFR pathway activation in the hematopoietic progenitors of the lymph gland. Interestingly, EGFR ligand signaling via exosomes has been reported in mammals where they promote tumorigenesis when released by cancer cells (106).

Previous data proposed that Spitz produced by PSC cells is involved in the differentiation of embryo-derived blood cells circulating in the hemolymph (the circulatory system of *Drosophila* larvae) into lamellocytes, although no mechanism was identified (39). Since EVs can circulate through biological fluids, both in *Drosophila* and in vertebrates, to mediate long-range inter-organ communication (56, 98, 107–109), one possibility is that PSC-derived exosomes could disseminate into the hemolymph to activate EGFR signaling in circulating blood cells, orchestrating hence a systemic immune response to wasp parasitism.

In the mammalian bone marrow, exosomes promote niche/HSPC intercellular communication, both in healthy and malignant conditions (6). For example, mesenchymal stem cell-derived exosomes can promote HSPC homing and survival or induce hematopoietic stem cell expansion. Inversely, in hematological malignancies, exosomes derived from leukemic cells can orchestrate a harmful dialogue contributing to disease development when they transport cargoes different from exosomes secreted by normal cells, such as oncoproteins and pro-inflammatory chemokines. However, due to the high cellular complexity of the vertebrate bone marrow niche (up to 28 cell types) (11), the methodological challenges of tracking EV source and destination in the bone marrow, and the lack of robust genetic tools to abolish EV release in a cell-specific manner, studying *in vivo* the role of exosomes in niche/HSPC communication has remained limited in mammals (110–113). Knowledge has mostly been retrieved *ex vivo*, using exosomes collected from body fluids or in primary and cancer cell lines. Likewise, *in vivo* investigation of matrix-degrading proteinases associated with exosomes in facilitating tumor invasion and metastases by ECM remodeling is complex (9, 84, 98). Here, we show that the *Drosophila* lymph gland provides a powerful model to study *in vivo* how exosomes produced by the niche control hematopoietic cell fate, using a variety of genetic and molecular tools available to trace and manipulate exosomes in a cell- and temporal-specific manner.

## Data Availability

The original contributions presented in the study are included in the article/**Supplementary Material**. Further inquiries can be directed to the corresponding author.

## Supplementary material

Supplementary Figure 1 Identification of exosomes released by PSC cells. **(A–B)** *Antp-Gal4*-driven expression of *UAS-mCD8-GFP* in PSC cells of late L2 larvae produces GFP fluorescent punctae in lymph gland anterior lobes both under normal **(A)** and parasitized **(B)** conditions. **(C)** mCD8-GFP EV index in lymph gland anterior lobes. Error bars represent SEM, t-test (Mann-Whitney nonparametric test): **p<0.01. (D-D”) Representative confocal image of *col>eGFP>CD63* lymph gland 3h post-parasitism immuno-stained for CD63. eGFP (green in D; white in D’) and CD63 (red in D; white in D”). Only rare eGFP fluorescent punctae (white arrowheads) are detectable in lymph gland anterior lobes. (E-E”) Representative confocal image of *col>CD63-GFP* lymph gland immuno-stained under non-permeabilizing conditions with an anti-CD63 antibody to detect exosome-surface exposed CD63 (CD63^ext^) (red in E, white in E”) 3h post-parasitism. CD63-GFP (green in E; white in E’). (E’,E”) Magnification of the white square drawn in **(E)** showing that most of PSC-derived CD63-GFP exosomes are co-stained for CD63^ext^ (arrowheads). **(F)** CD63-GFP exosome index in lymph gland anterior lobes at different time points after parasitism. Error bars represent SEM, t-test (Mann-Whitney nonparametric test): **p<0.01; ***p<0.001 and ns (not significant). **(G)** Distribution (%) of CD63-GFP exosomes in lymph gland anterior lobes. Exosomes are scored depending on their position relative to PSC cells. Proximal to distant positions are indicated by a color code. Note that the percentage of exosomes at most distant locations from the PSC (above red lines) increases between 1 to 4h post-parasitism compared to non-parasitized conditions. Error bars correspond to SEM. (H) Electron microscopy imaging of PSC cells in *col>HRP*-*CD8* lymph gland. HRP-positive PSC membranes are easily recognizable due to labeling with DAB deposits (black). nuc, nuclei. All experiments were repeated independently at least three times with similar results. Scale bars: (A, B, D-D”,E) 40 μm, (E’,E”,H) 5 μm.

Supplementary Figure 2 Blocking the release of exosomes from the niche impairs lamellocyte differentiation and wasp egg encapsulation. **(A)** mCD8-GFP EV index 3h post-parasitism in lymph gland anterior lobe expressing a different RNAi for Rab27 (*col>mCD8-GFP>Rab27-RNAi#2*). Error bars represent SEM, t-test (Mann-Whitney nonparametric test): ***p<0.001. **(B, C)** Confocal images (z-projections) of mCD8-GFP-positive EVs 3h post-parasitism in lymph gland anterior lobe in control larvae (*Gal80^ts^;col>colmCD8-GFP)* **(B)** and when dominant negative VPS4 is expressed in the PSC (*Gal80^ts^;col>colmCD8-GFP>VPS4^DN^*) **(C)**. **(D)** mCD8-GFP EV index 3h post-parasitism in lymph gland anterior lobes. Error bars represent SEM, t-test (Mann-Whitney nonparametric test): **p<0.01. **(E–H)** Ubiquitin immunodetection (red in E-H; white in E’-H’) 6h post parasitism in PSC cells (green in E–H). Ubiquitin-positive aggregates are detected in PSC cells of *Gal80^ts^;col>VPS4^DN^* larvae (F,F’) but not in *col>Rab27-KD* (H,H’). **(I)** Quantification (%) of wasp egg encapsulation. Box plots represent the mean of at least three biological replicates. Error bars correspond to SEM, *p<0.1; ***p<0.001 (Pearson’s Chi-squared test). **(J–K)** Representative confocal image of lamellocyte staining (β-integrin, white) 10h post-parasitism in control lymph gland (*col>)* **(J)** and when Rab27 is downregulated in PSC cells (*col>Rab27-RNAi*) (K). Arrows indicate lymph gland rupture at the onset of lamellocyte dispersal. (CT) cardiac tube. **(L)** Lamellocyte index. **p<0.01 (t-test; Mann-Whitney nonparametric test). Experiments were repeated independently at least three times with similar results and one experiment is shown. Scale bars: 40 μm.

Supplementary Figure 3 ROS level increase in the niche is an early response to wasp attack. **(A–B)** β-gal immunostaining (red in A,B; white in A’,B’) in *col>CD63-GFP* lymph glands expressing the *gstD-lacZ* transgene. An increase in ROS levels in PSC cells (GFP, green) is observed 2h post-parasitism. **(C, D)** Representative confocal image (single z section) of extracellular CD63 (CD63^ext^, white) at the surface of PSC cells 3h post-parasitism in control *col>CD63-GFP* **(C)** and *col>CD63-GFP>catalase* **(D)** lymph glands immunostained for CD63 under non-permeabilizing conditions. Yellow arrowheads: exosomes at PSC cell junctions. **(E)** Quantification of CD63^ext^ intensity per PSC in *col>CD63* lymph glands. Error bars represent SEM, ***p<0.001 t-test (Mann-Whitney nonparametric test). (F–G”) Detection of CD63-GFP exosomes (green in **F, G**; white in F’,G’) in progenitors (blue, DAPI) of *col>CD63-GFP* lymph glands expressing either mCherry-Rab5 under endogenous regulatory elements (red in F, white in F”), or *Ubi>Rab11-mCherry* (red in G, white in G”). Yellow circles indicate colocalization between CD63-positive exosomes and endogenous Rab5 or Rab11-positive endosomes. All experiments were repeated independently three times with similar results and one experiment is shown. Scale bars: (A–B’, F–G”) 40 μm, **(C, D)** 5 μm.

Supplementary Figure 4 The ECM surrounding the PSC is not degraded following parasitism, while TIMP expression in the niche impairs wasp egg encapsulation. (A-B’) Close up view (single confocal section) of the ECM around PSC cells in *col>CD63-mCherry* (PSC, blue) lymph gland expressing Vkg::GFP (magenta) under normal (non-parasitized) conditions (A,A’) and 3h post-parasitism (B,B’). (C-H’) Representative confocal image (single section) of *col>CD63-GFP* (PSC, blue) lymph gland anterior lobes immuno-stained for Trol (magenta in C-D’), αPS4 (magenta in E-F’) or Coracle (magenta in G-H’) under non-parasitized conditions (C,C’,E,E’,G,G’) and 3h post-parasitism (D,D’,F,F’,H,H’). Experiments were repeated independently at least three times with similar results. (I) Quantification (%) of wasp egg encapsulation. Box plots represent the mean of at least three biological replicates. Error bars correspond to SEM, ***p<0.001 (Pearson’s Chi-squared test). Scale bars: 40 μm.

Supplementary Figure 5 Mmp2 expression in the lymph gland is not affected 3h post-parasitism. Mmp1 expression in PSC cells is required for exosome spreading from the niche and wasp egg encapsulation. (A-B’) Representative confocal image (single z section) of Mmp2 immunostaining (red in **A, B**; white in A’,B’) in lymph gland anterior lobe of control larvae (*col>CD63-GFP*) without (A,A’) and 3h post-parasitism (B-B’). The PSC expresses CD63-GFP (green in A, B) and is outlined in white. (C–C’’) Close up view (single confocal section) of Mmp1 immunostaining (red in **C**; white in C’,C’’) in PSC cells in *col>CD63-GFP* lymph gland 3h post-parasitism. Mmp1 labels discrete intracellular CD63-positive punctae (white arrowheads in C, yellow in C’,C”). **(D, E)** Super resolution confocal imaging (z-projection) of CD63-GFP exosomes (white) in the ECM (Trol, magenta) 3h post-parasitism in *col>CD63-GFP* (control) (D) and *col>CD63-GFP>Mmp1-RNAi* **(E)** lymph glands. Note that scarce CD63-GFP exosomes (arrow) localize in the ECM adjacent to the PSC when Mmp1 expression is knocked down in PSC cells. (F–G’) Mmp1 immunostaining (red in F,G; white in F’,G’) 3h post-parasitism in control lymph glands (*col>mCD8-GFP*) (F,F’) and when Mmp1 is down-regulated in PSC cells (*col>mCD8-GFP>Mmp1-RNAi*) (G,G’). The PSC expresses GFP (green in **F, G**) and is outlined in white. Note that Mmp1 is expressed in lymph gland progenitors in both contexts. (Prog.) progenitors. **(H, I)** Super-resolution confocal imaging (single z section) of extracellular CD63 (CD63^ext^, white) at the surface of PSC cells in *col>CD63* (control) (H) and *col>CD63 >Mmp1^E225A^* **(I)** lymph glands 3h post-parasitism. **(J)** Quantification of CD63^ext^ intensity per PSC. Error bars represent SEM, **p<0.01 t-test (Mann-Whitney nonparametric test). Experiments were repeated independently at least three times with similar results. **(K)** Wasp egg encapsulation (%) when Mmp1 is knocked down in PSC cells using two different UAS-RNAi lines (*Antp>Mmp1-RNAi* and *Antp>Mmp1-RNAi#2*). Box plots represent mean of at least 3 biological replicates. Error bars correspond to SEM, ***p<0.001 (Pearson’s Chi-squared test). Scale bars: (A-B’,F-G’) 40 μm, (C-C’’,D,E, H,I) 5 μm.
