# Supplementary figures and images for: Niche-derived exosomes control Drosophila immune stress hematopoiesis
Source: Front Immunol. 2026 Jun 10;17:1824544. doi: 10.3389/fimmu.2026.1824544 (PMC13290576; doi:10.3389/fimmu.2026.1824544)

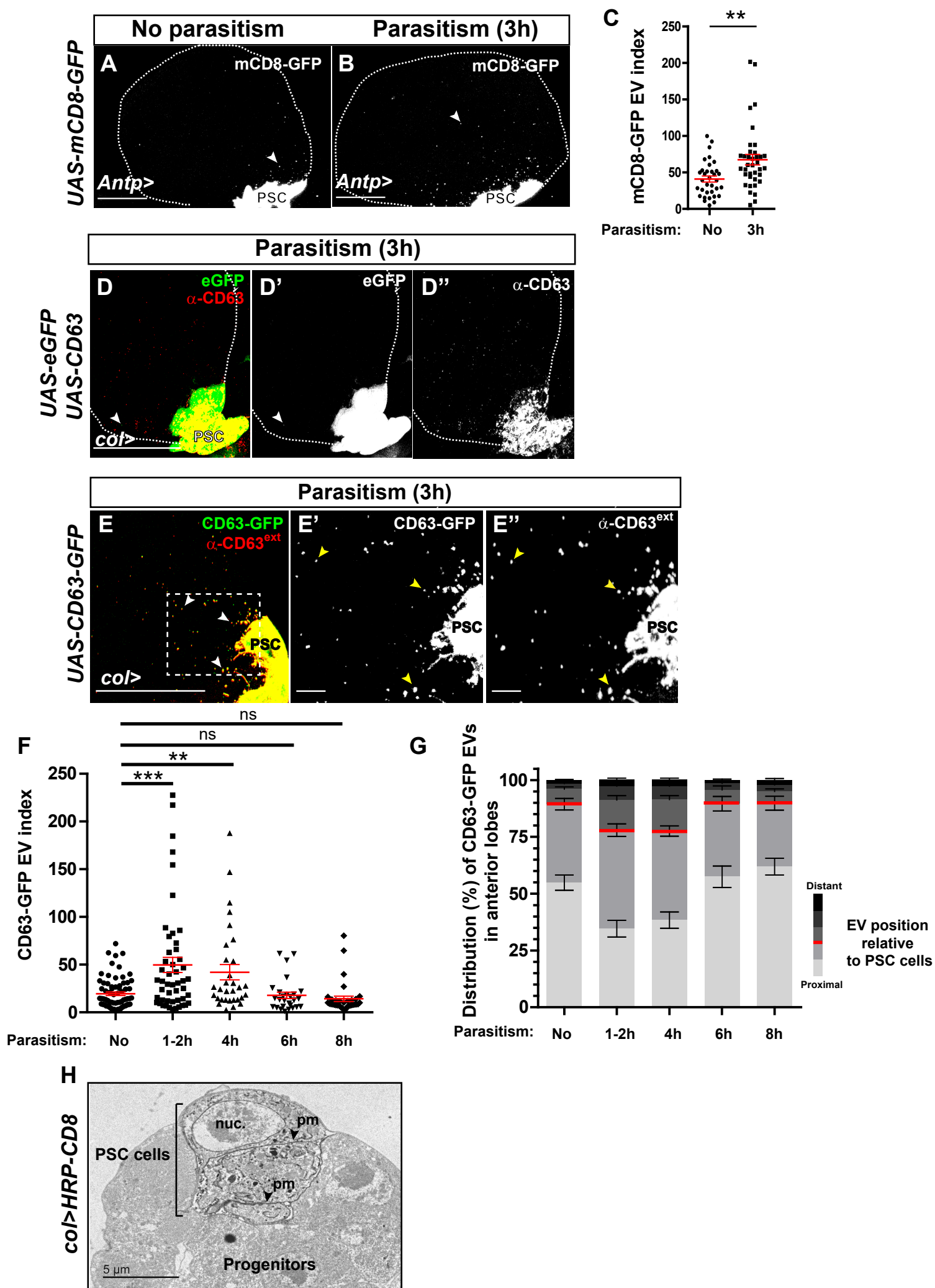

Figure 1 sup

Supplement: Supplementary Figure 1 — Identification of exosomes released by PSC cells. (A–B) Antp-Gal4-driven expression of UAS-mCD8-GFP in PSC cells of late L2 larvae produces GFP fluorescent punctae in lymph gland anterior lobes both under normal (A) and parasitized (B) conditions. (C) mCD8-GFP EV index in lymph gland anterior lobes. Error bars represent SEM, t-test (Mann-Whitney nonparametric test): **p<0.01. (D-D”) Representative confocal image of col>eGFP>CD63 lymph gland 3h post-parasitism immuno-stained for CD63. eGFP (green in D; white in D’) and CD63 (red in D; white in D”). Only rare eGFP fluorescent punctae (white arrowheads) are detectable in lymph gland anterior lobes. (E-E”) Representative confocal image of col>CD63-GFP lymph gland immuno-stained under non-permeabilizing conditions with an anti-CD63 antibody to detect exosome-surface exposed CD63 (CD63ext) (red in E, white in E”) 3h post-parasitism. CD63-GFP (green in E; white in E’). (E’,E”) Magnification of the white square drawn in (E) showing that most of PSC-derived CD63-GFP exosomes are co-stained for CD63ext (arrowheads). (F) CD63-GFP exosome index in lymph gland anterior lobes at different time points after parasitism. Error bars represent SEM, t-test (Mann-Whitney nonparametric test): **p<0.01; ***p<0.001 and ns (not significant). (G) Distribution (%) of CD63-GFP exosomes in lymph gland anterior lobes. Exosomes are scored depending on their position relative to PSC cells. Proximal to distant positions are indicated by a color code. Note that the percentage of exosomes at most distant locations from the PSC (above red lines) increases between 1 to 4h post-parasitism compared to non-parasitized conditions. Error bars correspond to SEM. (H) Electron microscopy imaging of PSC cells in col>HRP-CD8 lymph gland. HRP-positive PSC membranes are easily recognizable due to labeling with DAB deposits (black). nuc, nuclei. All experiments were repeated independently at least three times with similar results. Scale bars: (A, B, D-D”,E) 40 μm, [file DataSheet1.pdf]

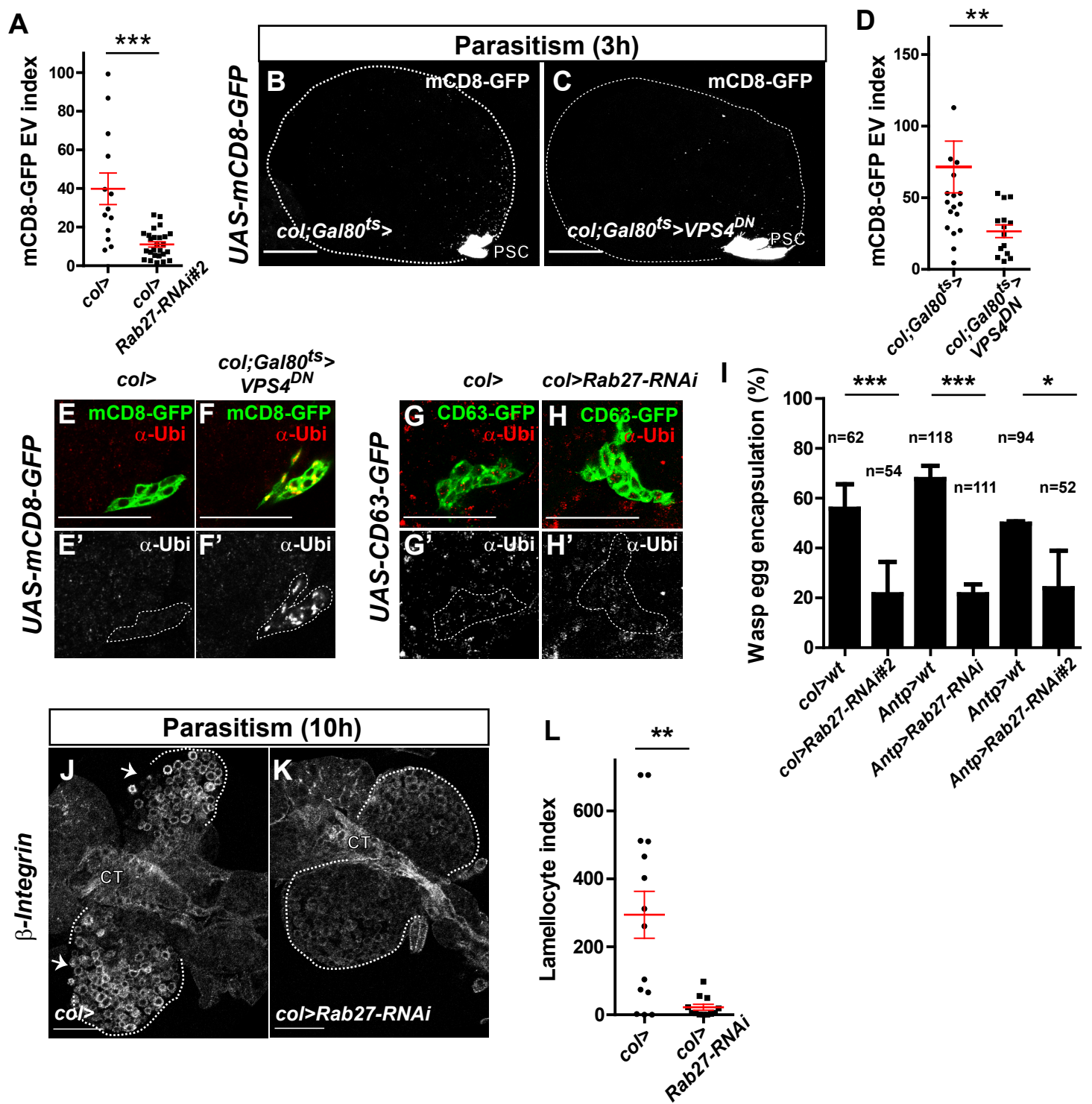

Figure 2 sup

Supplement: Supplementary Figure 2 — Blocking the release of exosomes from the niche impairs lamellocyte differentiation and wasp egg encapsulation. (A) mCD8-GFP EV index 3h post-parasitism in lymph gland anterior lobe expressing a different RNAi for Rab27 (col>mCD8-GFP>Rab27-RNAi#2). Error bars represent SEM, t-test (Mann-Whitney nonparametric test): ***p<0.001. (B, C) Confocal images (z-projections) of mCD8-GFP-positive EVs 3h post-parasitism in lymph gland anterior lobe in control larvae (Gal80ts;col>colmCD8-GFP) (B) and when dominant negative VPS4 is expressed in the PSC (Gal80ts;col>colmCD8-GFP>VPS4DN) (C). (D) mCD8-GFP EV index 3h post-parasitism in lymph gland anterior lobes. Error bars represent SEM, t-test (Mann-Whitney nonparametric test): **p<0.01. (E–H) Ubiquitin immunodetection (red in E-H; white in E’-H’) 6h post parasitism in PSC cells (green in E–H). Ubiquitin-positive aggregates are detected in PSC cells of Gal80ts;col>VPS4DN larvae (F,F’) but not in col>Rab27-KD (H,H’). (I) Quantification (%) of wasp egg encapsulation. Box plots represent the mean of at least three biological replicates. Error bars correspond to SEM, *p<0.1; ***p<0.001 (Pearson’s Chi-squared test). (J–K) Representative confocal image of lamellocyte staining (β-integrin, white) 10h post-parasitism in control lymph gland (col>) (J) and when Rab27 is downregulated in PSC cells (col>Rab27-RNAi) (K). Arrows indicate lymph gland rupture at the onset of lamellocyte dispersal. (CT) cardiac tube. (L) Lamellocyte index. **p<0.01 (t-test; Mann-Whitney nonparametric test). Experiments were repeated independently at least three times with similar results and one experiment is shown. Scale bars: 40 μm. [file DataSheet2.pdf]

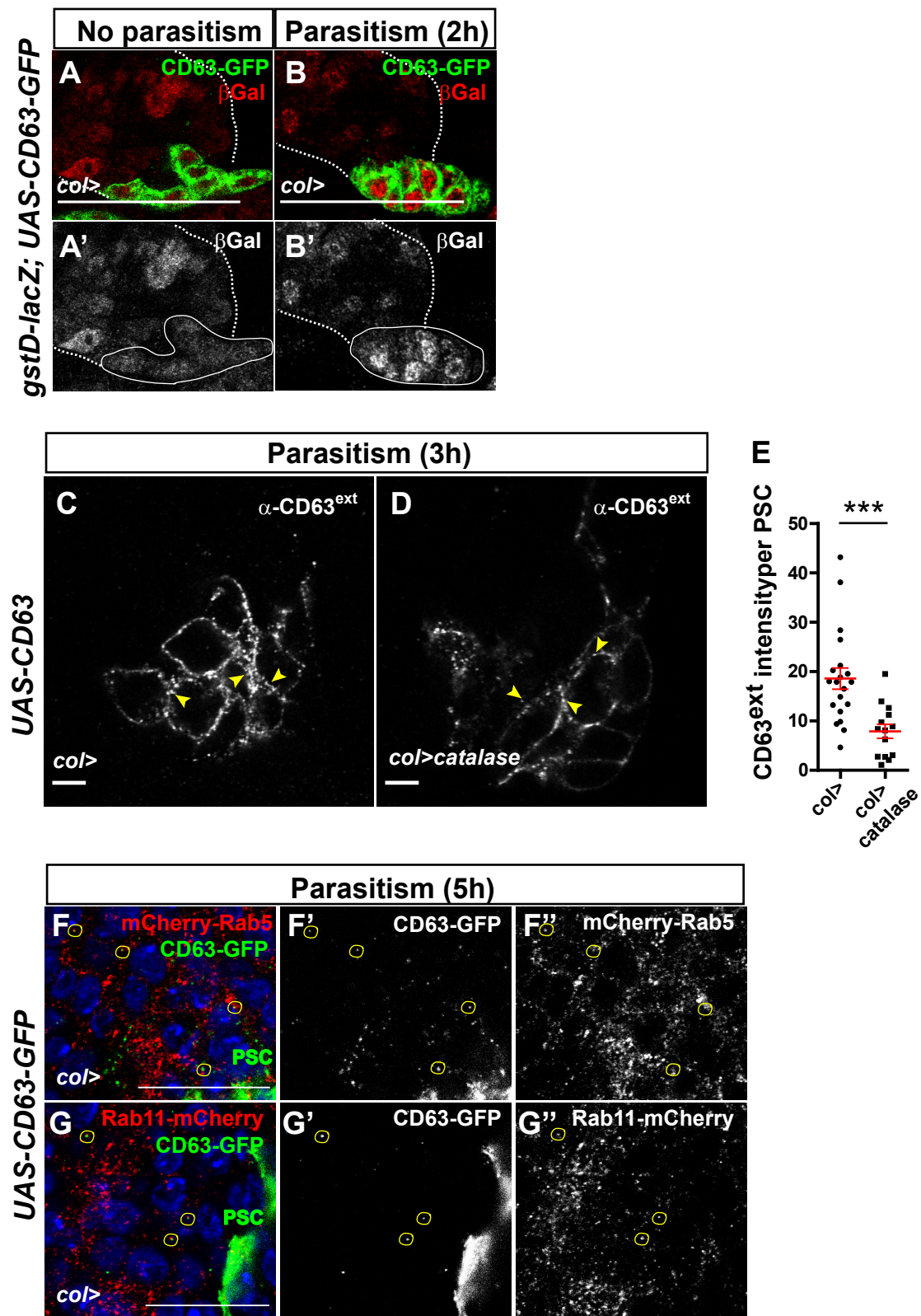

Figure 3 sup

Supplement: Supplementary Figure 3 — ROS level increase in the niche is an early response to wasp attack. (A–B) β-gal immunostaining (red in A,B; white in A’,B’) in col>CD63-GFP lymph glands expressing the gstD-lacZ transgene. An increase in ROS levels in PSC cells (GFP, green) is observed 2h post-parasitism. (C, D) Representative confocal image (single z section) of extracellular CD63 (CD63ext, white) at the surface of PSC cells 3h post-parasitism in control col>CD63-GFP (C) and col>CD63-GFP>catalase (D) lymph glands immunostained for CD63 under non-permeabilizing conditions. Yellow arrowheads: exosomes at PSC cell junctions. (E) Quantification of CD63ext intensity per PSC in col>CD63 lymph glands. Error bars represent SEM, ***p<0.001 t-test (Mann-Whitney nonparametric test). (F–G”) Detection of CD63-GFP exosomes (green in F, G; white in F’,G’) in progenitors (blue, DAPI) of col>CD63-GFP lymph glands expressing either mCherry-Rab5 under endogenous regulatory elements (red in F, white in F”), or Ubi>Rab11-mCherry (red in G, white in G”). Yellow circles indicate colocalization between CD63-positive exosomes and endogenous Rab5 or Rab11-positive endosomes. All experiments were repeated independently three times with similar results and one experiment is shown. Scale bars: (A–B’, F–G”) 40 μm, (C, D) 5 μm. [file DataSheet3.pdf]

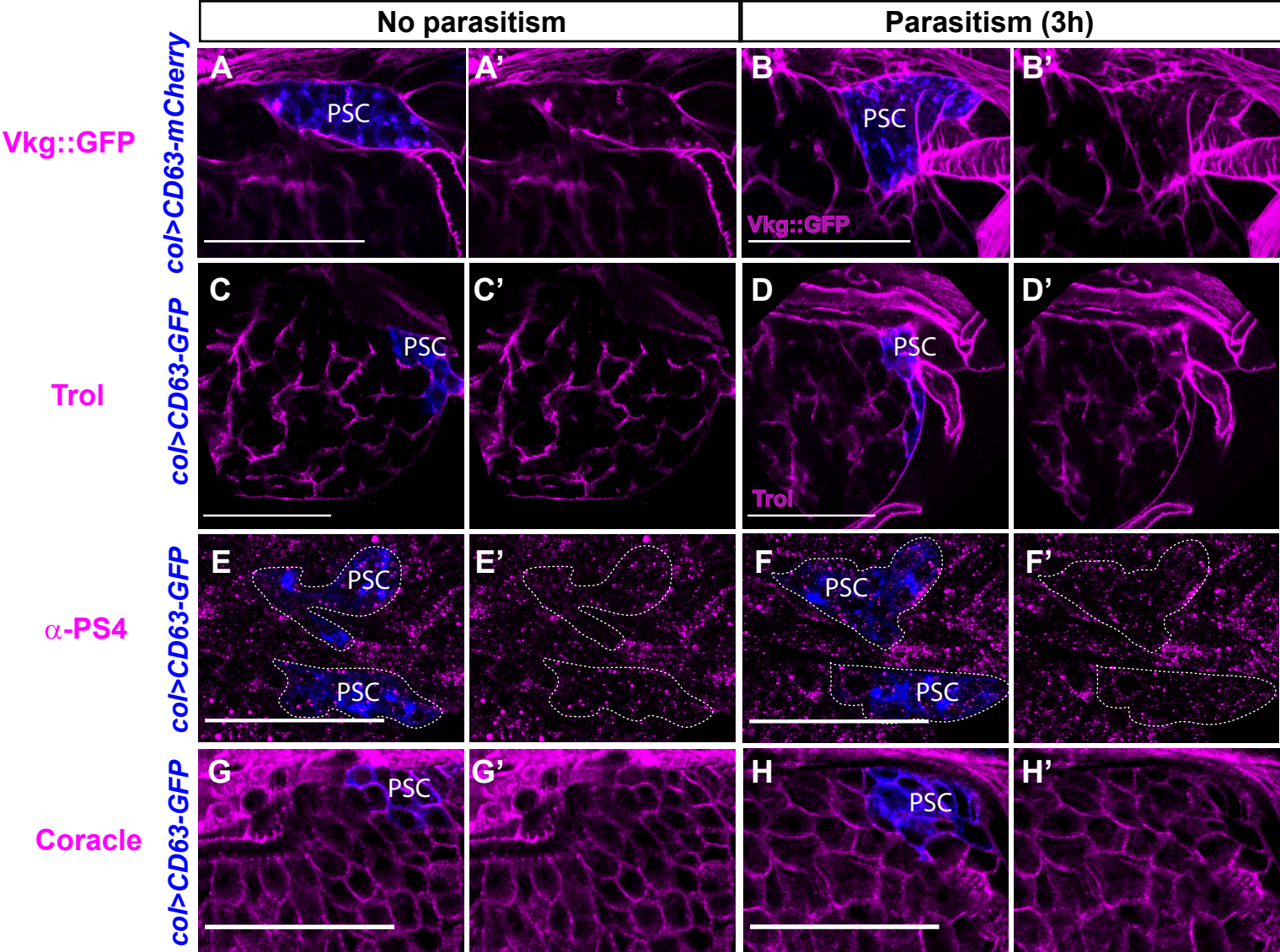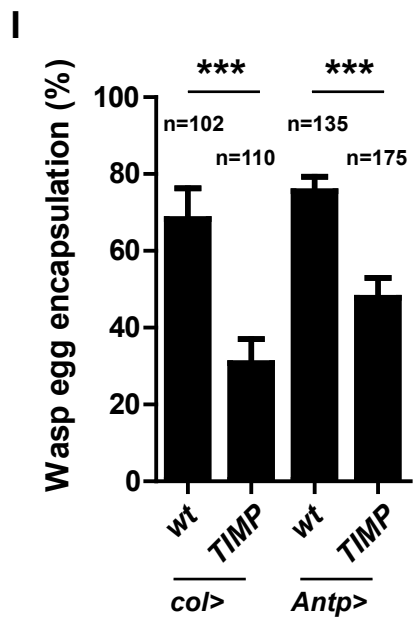

Figure 4 sup

Supplement: Supplementary Figure 4 — The ECM surrounding the PSC is not degraded following parasitism, while TIMP expression in the niche impairs wasp egg encapsulation. (A-B’) Close up view (single confocal section) of the ECM around PSC cells in col>CD63-mCherry (PSC, blue) lymph gland expressing Vkg::GFP (magenta) under normal (non-parasitized) conditions (A,A’) and 3h post-parasitism (B,B’). (C-H’) Representative confocal image (single section) of col>CD63-GFP (PSC, blue) lymph gland anterior lobes immuno-stained for Trol (magenta in C-D’), αPS4 (magenta in E-F’) or Coracle (magenta in G-H’) under non-parasitized conditions (C,C’,E,E’,G,G’) and 3h post-parasitism (D,D’,F,F’,H,H’). Experiments were repeated independently at least three times with similar results. (I) Quantification (%) of wasp egg encapsulation. Box plots represent the mean of at least three biological replicates. Error bars correspond to SEM, ***p<0.001 (Pearson’s Chi-squared test). Scale bars: 40 μm. [file DataSheet4.pdf]

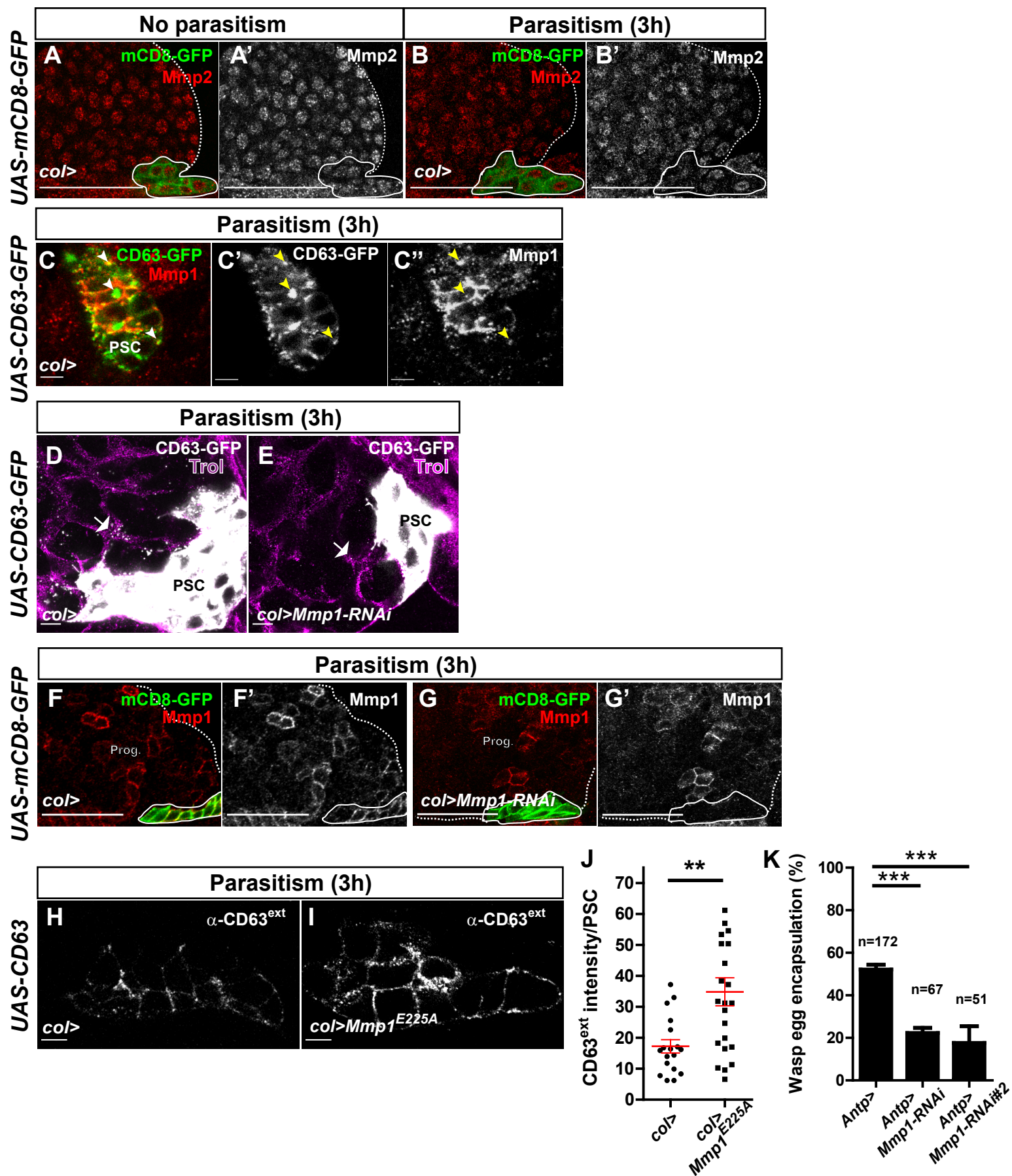

Supplement: Supplementary Figure 5 — Mmp2 expression in the lymph gland is not affected 3h post-parasitism. Mmp1 expression in PSC cells is required for exosome spreading from the niche and wasp egg encapsulation. (A-B’) Representative confocal image (single z section) of Mmp2 immunostaining (red in A, B; white in A’,B’) in lymph gland anterior lobe of control larvae (col>CD63-GFP) without (A,A’) and 3h post-parasitism (B-B’). The PSC expresses CD63-GFP (green in A, B) and is outlined in white. (C–C’’) Close up view (single confocal section) of Mmp1 immunostaining (red in C; white in C’,C’’) in PSC cells in col>CD63-GFP lymph gland 3h post-parasitism. Mmp1 labels discrete intracellular CD63-positive punctae (white arrowheads in C, yellow in C’,C”). (D, E) Super resolution confocal imaging (z-projection) of CD63-GFP exosomes (white) in the ECM (Trol, magenta) 3h post-parasitism in col>CD63-GFP (control) (D) and col>CD63-GFP>Mmp1-RNAi (E) lymph glands. Note that scarce CD63-GFP exosomes (arrow) localize in the ECM adjacent to the PSC when Mmp1 expression is knocked down in PSC cells. (F–G’) Mmp1 immunostaining (red in F,G; white in F’,G’) 3h post-parasitism in control lymph glands (col>mCD8-GFP) (F,F’) and when Mmp1 is down-regulated in PSC cells (col>mCD8-GFP>Mmp1-RNAi) (G,G’). The PSC expresses GFP (green in F, G) and is outlined in white. Note that Mmp1 is expressed in lymph gland progenitors in both contexts. (Prog.) progenitors. (H, I) Super-resolution confocal imaging (single z section) of extracellular CD63 (CD63ext, white) at the surface of PSC cells in col>CD63 (control) (H) and col>CD63 >Mmp1E225A (I) lymph glands 3h post-parasitism. (J) Quantification of CD63ext intensity per PSC. Error bars represent SEM, **p<0.01 t-test (Mann-Whitney nonparametric test). Experiments were repeated independently at least three times with similar results. (K) Wasp egg encapsulation (%) when Mmp1 is knocked down in PSC cells using two different UAS-RNAi lines (Antp>Mmp1-RNAi and Antp>Mmp1-RNAi#2). Box plots repr [file DataSheet5.pdf]
