# Supplementary material for: Niche-derived exosomes control Drosophila immune stress hematopoiesis
Source: Front Immunol. 2026 Jun 10;17:1824544. doi: 10.3389/fimmu.2026.1824544 (PMC13290576; doi:10.3389/fimmu.2026.1824544)
Supplement: Supplementary file 6 [file DataSheet6.docx]

**Drosophila genetics: Fly crosses for each figure**

**Figure 1A-C:** *col-Gal4*, *UAS-dicer*, *UAS-mCD8-GFP* crossed with *w^1118^* (control), **D-F:** *col-Gal4*, *UAS-dicer* crossed with *UAS-CD63-GFP,* **G-G”**; *col-Gal4*, *UAS-dicer*, *UAS-mCD8-GFP* crossed with *UAS-CD63,* **H-J:** *col-Gal4*, *UAS-dicer* crossed with *UAS-CD63.* **K-M:** *col-Gal4*, *UAS-dicer crossed with UAS-HRP-CD8.*

**Sup. Figure 1A-C:** Antp*-Gal4*, *UAS-dicer*, *UAS-mCD8-GFP* crossed with *w^1118^* (control), **D-D”:** *col-Gal4*, *UAS-dicer*, *UAS-eGFP* crossed with *UAS-CD63,* ***E-E”:*** *col-Gal4*, *UAS-dicer* crossed with *UAS-CD63-GFP,* **F-G:** *col-Gal4*, *UAS-dicer* crossed with *UAS-CD63-GFP*, **H:** *col-Gal4*, *UAS-dicer crossed with UAS-HRP-CD8.*

**Figure 2A-C:** *col-Gal4*, *UAS-dicer*, *UAS-CD63-GFP* crossed with *w^1118^* (control) or *UAS-Rab27-RNAi (31887)*, **D-E:** *col-Gal4*, *UAS-dicer*, *UAS-mCD8-GFP* crossed with *w^1118^* (control) or *UAS-Rab27-RNAi (31887)*, **F-H:** *col-Gal4*, *UAS-dicer*, DoxA3::GFP crossed with *w^1118^* (control) or *UAS-Rab27-RNAi (31887)*.

**Sup. Figure 2A:** *col-Gal4*, *UAS-dicer*, *UAS-CD63-GFP* crossed with *w^1118^* (control) or *UAS-Rab27-RNAi#2 (*50537), **B-F:** *col-Gal4*, *UAS-dicer, UAS-mCD8-GFP, tub-Gal80^ts^* crossed with *w^1118^* (control) or *UAS-VPS4^DN^*, **G-H:** *col-Gal4*, *UAS-dicer*, *UAS-CD63-GFP* crossed with *w^1118^* (control) or *UAS-Rab27-RNAi (31887)*. **I:** *col-Gal4*, *UAS-dicer*, *UAS-mCD8-GFP* crossed with *w^1118^* (control) or *UAS-Rab27-RNAi#2 (*50537), *Antp-Gal4*, *UAS-dicer*, *UAS-mCD8-GFP* crossed with *w^1118^* (control) or *UAS-Rab27-RNAi (31887) or UAS-Rab27-RNAi#2 (*50537). **J-L:** *col-Gal4*, *UAS-dicer*, *UAS-mCD8-GFP* crossed with *w^1118^* (control) or *UAS-Rab27-RNAi (31887).*

**Figure 3A-C:** *col-Gal4*, *UAS-dicer*, *UAS-CD63-GFP* crossed with *w^1118^* (control) or *UAS-catalase,* **D-F:** *col-Gal4*, *UAS-dicer*, *UAS-mCD8-GFP* crossed with *w^1118^* (control) or *UAS-Rab27-RNAi (31887)*, **G-G”:** *Antp-Gal4*, *UAS-dicer, tub-Gal80^ts^, UAS-CD63-mCherry* crossed with *UAS-cSpi-GFP*, **H:** *Antp-Gal4,* *UAS-dicer, tub-Gal80^ts^* crossed with *w^1118^* (control) or *UAS-Rab27-RNAi (31887) or UAS-cSpi or UAS-Rab27-RNAi (31887), UAS-cSpi.*

**Sup. Figure 3A-B':** *col-Gal4*, *UAS-dicer, gstD-lacZ* crossed with *UAS-CD63-GFP,* **C-E:** *col-Gal4*, *UAS-dicer*, *UAS-CD63 crossed with w^1118^* (control) or *UAS-catalase.*

**F-F”:** *col-Gal4*, *UAS-dicer*, *UAS-CD63-GFP* crossed with *mCherry-Rab5,* **G-G”:** *col-Gal4*, *UAS-dicer*, *UAS-CD63-GFP* crossed with *Ubiquitin-Rab11-mCherry*.

**Figure 4A:** *col-Gal4*, *UAS-dicer, Vkg::GFP crossed with UAS-CD63,* **B:** *col-Gal4*, *UAS-dicer* crossed with *UAS-CD63-GFP,* ***C-E:*** *col-Gal4*, *UAS-dicer*, *UAS-CD63-GFP* crossed with *w^1118^* (control) or *UAS-TIMP,* ***F-H:*** *col-Gal4*, *UAS-dicer*, *UAS-CD63 crossed with w^1118^* (control) or *UAS-TIMP,* ***I-K:*** *col-Gal4*, *UAS-dicer*, *UAS-mCD8-GFP* crossed with *w^1118^* (control) or *UAS-TIMP.*

**Sup.Figure 4A-B’:** *col-Gal4*, *UAS-dicer*, *Vkg::GFP* *crossed with UAS-CD63-mCherry,* ***C-H’****: col-Gal4*, *UAS-dicer*, *UAS-CD63-GFP* crossed with *w^1118^* (control), **I:** *col-Gal4*, *UAS-dicer*, *UAS-mCD8-GFP* crossed with *w^1118^* (control) or *UAS-TIMP.*

**Figure 5A-C’:** *col-Gal4*, *UAS-dicer*, *UAS-CD63-GFP* crossed with *w^1118^* (control) or *UAS-catalase,* ***D-G****: col-Gal4*, *UAS-dicer*, *UAS-CD63-GFP* crossed with *w^1118^* (control) or *UAS-Mmp1-RNAi or UAS-Mmp1-RNAi#2 (*31489), **H-K:** *col-Gal4*, *UAS-dicer*, *UAS-CD63-GFP* crossed with *w^1118^* (control) or *UAS-Mmp1^E225A^* or *UAS-Mmp1,* **L-L”:** *col-Gal4*, *UAS-dicer*, *UAS-CD63-GFP* crossed with *w^1118^* (control).

**Sup.Figure 5A-B’:** *col-Gal4*, *UAS-dicer*, *UAS-mCD8-GFP* crossed with *w^1118^* (control), **C-C”:** *col-Gal4*, *UAS-dicer*, *UAS-CD63-GFP* crossed with *w^1118^* (control), **D-E:** *col-Gal4*, *UAS-dicer*, *UAS-CD63-GFP* crossed with *w^1118^* (control) or *UAS-Mmp1-RNAi*, **F-G’:** *col-Gal4*, *UAS-dicer*, *UAS-mCD8-GFP* crossed with *w^1118^* (control) or *UAS-Mmp1-RNAi*, **H-J:** *col-Gal4*, *UAS-dicer, UAS-CD63* *crossed with w^1118^* (control) or *UAS- Mmp1^E225A^*, **K:** *Antp-Gal4*, *UAS-dicer* *crossed with w^1118^* (control) or *UAS-Mmp1-RNAi or UAS-Mmp1-RNAi#2 (*31489).
